# Endoscopic ultrasonography-based intratumoral and peritumoral machine learning radiomics analyses for distinguishing insulinomas from non-functional pancreatic neuroendocrine tumors

**DOI:** 10.3389/fendo.2024.1383814

**Published:** 2024-06-17

**Authors:** Shuangyang Mo, Cheng Huang, Yingwei Wang, Huaying Zhao, Wenhong Wu, Haixing Jiang, Shanyu Qin

**Affiliations:** ^1^ Gastroenterology Department, Liuzhou People’s Hospital Affiliated to Guangxi Medical University, Liuzhou, China; ^2^ Gastroenterology Department, The First Affiliated Hospital of Guangxi Medical University, Nanning, China; ^3^ Oncology Department, Liuzhou People’s Hospital Affiliated to Guangxi Medical University, Liuzhou, China

**Keywords:** pancreatic neuroendocrine tumors, insulinomas, peritumoral, endoscopic ultrasonography, radiomics, machine learning, nomogram

## Abstract

**Objectives:**

To develop and validate radiomics models utilizing endoscopic ultrasonography (EUS) images to distinguish insulinomas from non-functional pancreatic neuroendocrine tumors (NF-PNETs).

**Methods:**

A total of 106 patients, comprising 61 with insulinomas and 45 with NF-PNETs, were included in this study. The patients were randomly assigned to either the training or test cohort. Radiomics features were extracted from both the intratumoral and peritumoral regions, respectively. Six machine learning algorithms were utilized to train intratumoral prediction models, using only the nonzero coefficient features. The researchers identified the most effective intratumoral radiomics model and subsequently employed it to develop peritumoral and combined radiomics models. Finally, a predictive nomogram for insulinomas was constructed and assessed.

**Results:**

A total of 107 radiomics features were extracted based on EUS, and only features with nonzero coefficients were retained. Among the six intratumoral radiomics models, the light gradient boosting machine (LightGBM) model demonstrated superior performance. Furthermore, a peritumoral radiomics model was established and evaluated. The combined model, integrating both the intratumoral and peritumoral radiomics features, exhibited a comparable performance in the training cohort (AUC=0.876) and achieved the highest accuracy in predicting outcomes in the test cohorts (AUC=0.835). The Delong test, calibration curves, and decision curve analysis (DCA) were employed to validate these findings. Insulinomas exhibited a significantly smaller diameter compared to NF-PNETs. Finally, the nomogram, incorporating diameter and radiomics signature, was constructed and assessed, which owned superior performance in both the training (AUC=0.929) and test (AUC=0.913) cohorts.

**Conclusion:**

A novel and impactful radiomics model and nomogram were developed and validated for the accurate differentiation of NF-PNETs and insulinomas utilizing EUS images.

## Introduction

Pancreatic neuroendocrine tumors (PNETs) are rare tumors that originate from neuroendocrine cells in the pancreatic islet tissues, accounting for approximately 1-3% of all pancreatic neoplasms (1, 2). They present an extreme degree of heterogeneity in clinic pathological characteristics and prognosis (3, 4) and are broadly classified as functional PNETs (F-PNETs) and non-functional PNETs (NF-PNETs) depending on whether the evidence of hormone-producing (5, 6). Compared to NF-PNETs, F-PNETs have the capability to secrete various hormones or peptides such as insulin, gastrin, vasoactive intestinal peptide (VIP), glucagon, and somatostatin, resulting in distinct symptoms. Among these, insulinomas are the most prevalent subtype of F-PNETs, causing recurrent hypoglycemia as a consequence of persistent endogenous hyperinsulinism (7, 8). Patients with insulinomas often face challenges in diagnosis, as the diverse clinical presentations, nonspecific biochemical tests, and lack of a specific clinical diagnostic model can lead to misdiagnosis for prolonged periods (9–12). Additionally, distinguishing insulinoma from NF-PNETs in the early stages presents a further diagnostic challenge (13).

PNETs exhibit a wide range of biological behaviors, from low-grade malignancy to highly aggressive tumors (14). NF-PNETs, which are the predominant type of PNETs, often remain asymptomatic for extended periods and have been associated with worse prognoses compared to F-PNETs (15, 16). Presently, there is a lack of consensus and controversy regarding the most effective treatment approach for both NF-PNETs and insulinomas. However, guidelines from the European Neuroendocrine Tumor Society and the National Comprehensive Cancer Network suggest that asymptomatic NF-PNETs measuring less than 2cm may be safely observed without active surgical intervention (17, 18). In contrast, current guidelines generally advocate for surgical intervention in the case of insulinomas, while somatostatin analogs are increasingly being utilized for the treatment of well-differentiated, low-grade F-PNETs (19, 20). Consequently, the timely and precise diagnosis and prognostication of both NF-PNETs and insulinomas are of paramount importance in guiding treatment strategies.

The prevalence of PNETs has been on the rise in recent years, primarily attributed to the progress and utilization of diverse imaging techniques and modalities, including multidetector computerized tomography (MDCT), magnetic resonance imaging (MRI), and endoscopic ultrasonography (EUS) (21–24). The identification and classification of NF-PNETs and F-PNETs before surgery present a significant challenge, primarily relying on hormonal symptoms. While F-PNETs can secrete hormones, some patients may exhibit rare or mild endocrine symptoms before metastasis (25, 26). We propose that using imaging modalities could facilitate the identification of NF-PNETs and insulinomas, thereby aiding in therapeutic decision-making. However, the effectiveness of imaging modalities in improving the predictive accuracy of NF-PNETs and insulinomas remains unreported and unvalidated.

EUS is extensively utilized in diagnosing PNETs and is widely acknowledged as one of the most precise imaging modalities for pancreatic diseases owing to its capacity to generate high-resolution images of pancreatic lesions (27). Moreover, EUS is considered the preferred imaging modality in cases where alternative non-invasive imaging techniques yield negative results, as recommended by the consensus guidelines of the European Neuroendocrine Tumor Society (ENETS) in 2023 (12). The EUS method has been found to exhibit greater efficacy in detecting PNETs than CT and MRI, particularly in the case of small lesions (28). However, the current differentiation of pancreatic masses using EUS primarily relies on macroscopic anatomical imaging characteristics, leading to insufficient specificity and susceptibility to subjective interpretation by endoscopists.

Integrating radiomics and machine learning strategies has shown promising results in the differential diagnosis and prognosis prediction of various cancers (29). Radiomics facilitates extracting and analyzing numerous objective and internal image features using high-throughput techniques (30). Previous studies have successfully applied radiomics techniques to CT, MRI, and ultrasonography (US) for the diagnosis and prognostication of PNETs, highlighting their exceptional efficiency (31–33). Furthermore, previous studies have provided evidence to support a strong correlation between the radiomics features of the peritumoral region and various tumor-related factors, including diagnostic accuracy, pathological characteristics, and prognostic indicators (34–36).

However, the efficacy of radiomics approaches based on EUS in differentiating insulinomas from NF-PNETs is still uncertain, despite the recognition of EUS as a superior imaging technique. Given existing knowledge, we employed various commonly used machine learning algorithms to develop and verify a robust radiomics model utilizing intratumoral and peritumoral radiomics characteristics, to accurately distinguish insulinomas from NF-PNETs.

## Materials and methods

### Study population

This retrospective study obtained approval from the institutional ethics review board of the First Affiliated Hospital of Guangxi Medical University (No. 2023-K346-01, 2023-12-29), which granted a waiver for patient approval or signed informed consent for the review of medical images and clinical information. A total of 106 patients diagnosed with pancreatic tumors were selected for this research, comprising 61 patients with F-PNETs (all of which were insulinomas) and 45 patients with NF-PNETs who underwent pancreatic surgery or EUS-FNA at our institution from May 2012 to October 2023. The inclusion and exclusion criteria are delineated as follows.

The patients included in the study met the following criteria (1): they underwent a thorough preoperative contrast-enhanced CT and EUS scan of the pancreas (2); they were confirmed with either insulinomas or NF-PNET based on pathological examination and immunohistochemistry of tissue samples following surgical resection or EUS-FNA (3); complete and clear EUS images were available before the patient’s preoperative or pathological biopsies; and (4) patients who had not received any chemotherapy or radiotherapy before undergoing EUS. The patients excluded from the study met the following criteria (1): inability to display the entire lesion (2); significant motion artifacts or noticeable noise in the images; and (3) the presence of other types of tumors. The patients who were registered were randomly assigned to either a training cohort or a test cohort, with a ratio of 7:3.

**Figure 1:**
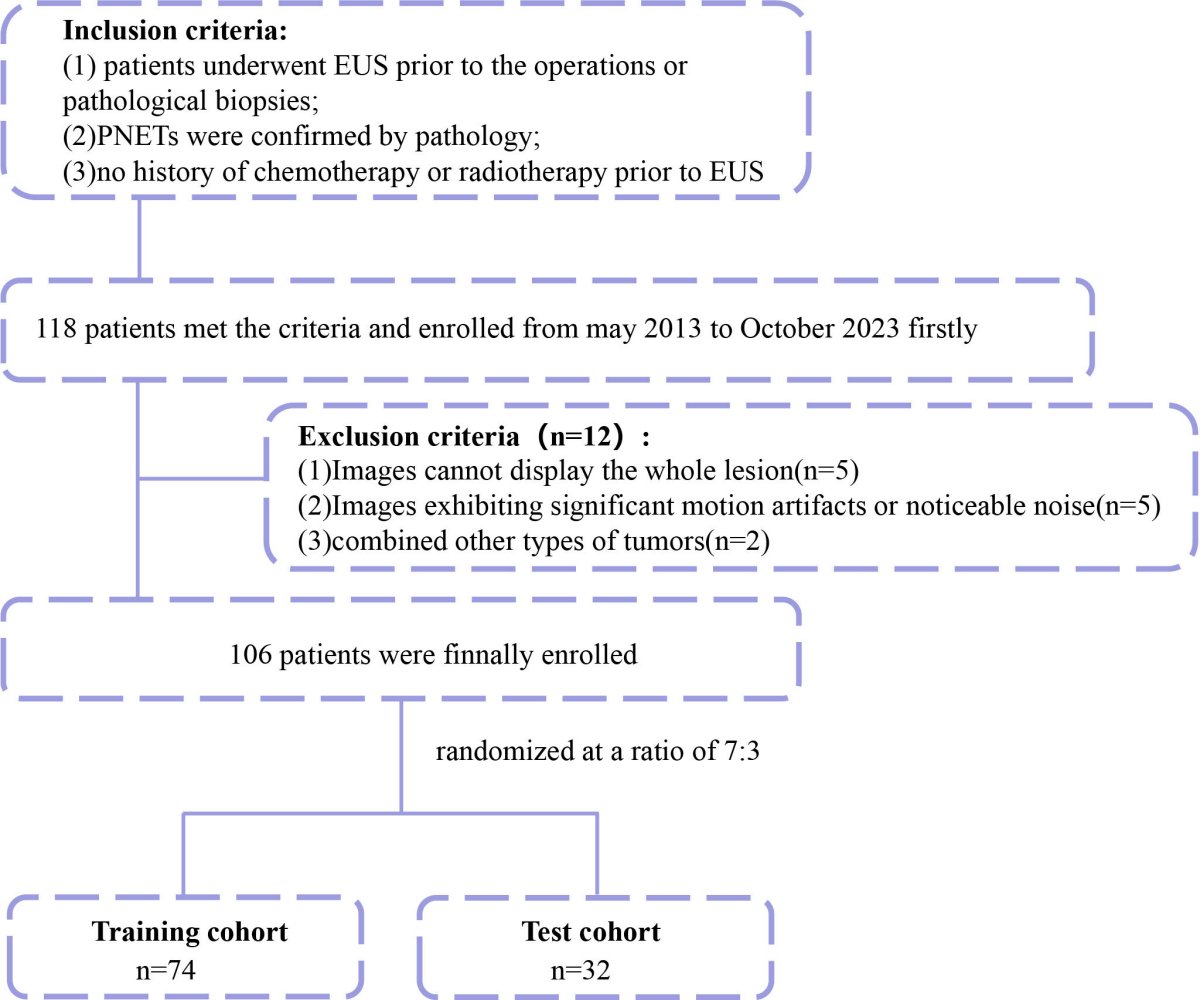
Flowchart for enrolling the study population.

The process of enrolling the study population is illustrated in **Figure 1**. This study involved a retrospective analysis of various clinical features, including age, gender, location of the pancreatic mass, echo characteristics, uniformity of the echo, maximum diameter, shape, margin characteristics, the presence of calcifications or cystic degeneration, and pathological diagnosis. Finally, any features that showed significant differences between patients with insulinomas and NF-PNETs were retained for the further construction of the nomogram.

### EUS image acquisition

The standard dynamic EUS procedure utilized the SU-9000 device (FUJIFILM, Japan) and the EU-ME2 device (Olympus, Japan). All electrocautery unit (ESU) image acquisition procedures were consistently performed by a highly experienced EUS specialist with a track record of over 12000 EUS practices. Meticulous scanning of the entire pancreatic region resulted in high-quality images of the pancreatic lesions. These images were consistently standardized with a window width of 250 Hounsfield units (HU) and a window level of 125 HU. The imaging records were obtained by retrieving data from our institutional Picture Archiving and Communication System (PACS).

### ROI delineation

**Figure 2:**
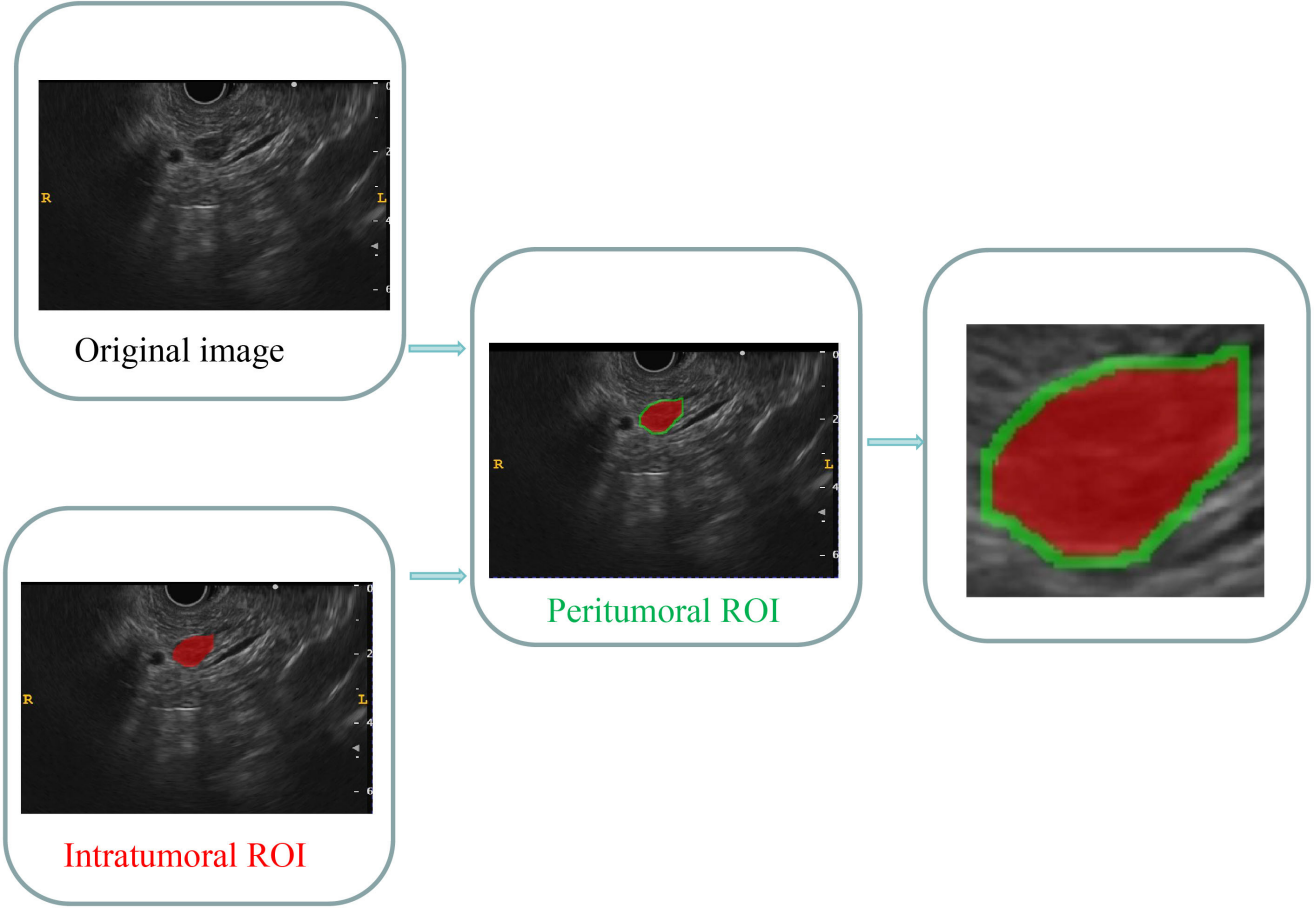
Comprehensive graph of the intratumoral and peritumoral ROIs. The red region indicates the “intratumoral ROI”; the green region indicates the “peritumoral ROI”. ROI, region of interest.

The images were stored in the Digital Imaging and Communications in Medicine (DICOM) format. Two specialists in EUS, each with 4 and 6 years of experience, manually segmented the intratumoral region of interest (ROI) using ITK-SNAP software (version 3.8.1, available at http://www.itksnap.org). In cases where conflicts arose between the two specialists, a dialog and consensus approach were employed. Both specialists were unaware of the pathological outcomes. The lesions were captured layer by layer along the margins on conventional EUS images, excluding adjacent normal tissue, vessels, bile ducts, and pancreatic ducts. The acquisition of the peritumoral ROI was achieved by employing a conventional morphological dilation technique using the ITK-SNAP software. This process involved expanding the delineation of the intratumoral ROI by 3 mm. Subsequently, three distinct ROI images were selected for each EUS image, namely an intratumoral ROI, a peritumoral ROI, and a combined ROI encompassing both the intratumoral and peritumoral ROIs. A comprehensive illustration depicting the procedure for acquiring the ROIs is presented in **Figure 2**.

Standardization techniques were implemented to preprocess the images and data, ensuring the reproducibility of the findings. The intraclass correlation coefficient (ICC) was employed to evaluate the replicability between observers and within observers. A cohort of 30 patients, consisting of 20 individuals with insulinomas and 10 with NF-PNETs, was randomly selected for inclusion. Following a one-week interval, the same EUS specialists conducted intratumoral ROI segmentation again. A threshold of an ICC value greater than 0.8 was established to indicate a significant level of agreement.

### Radiomics feature extraction

The categorization of handcrafted features can be delineated into three discrete groups, namely geometric, intensity, and textural. Geometric features are concerned with the three-dimensional morphological characteristics of tumors. Intensity features encompass the statistical dispersion of voxel intensities within the tumor in the first order. Conversely, textural features elucidate patterns and higher-order spatial distributions of intensities. This article utilized multiple methodologies, including the gray level co-occurrence matrix (GLCM), gray level run length matrix (GLRLM), gray level size zone matrix (GLSZM), and neighborhood gray-level difference matrix (NGTDM), to extract texture features. The extraction of radiomics features from the intratumoral and peritumoral regions of interest (ROIs) was conducted separately. The extraction and screening of radiomics features were performed using PyRadiomics, an internal feature analysis program, which facilitated the extraction of all handcrafted features. Additionally, the radiomics features of the combined ROIs were obtained by integrating the features extracted from both the intratumoral and peritumoral ROIs. The processes of extracting radiomics features followed the Image Biomarker Standardization Initiative (IBSI) (37).

### Radiomics feature selection

A Mann-Whitney U test was performed to screen features in both the training and test cohorts. Only radiomics features with a significance level of p<0.05 were retained for further analysis.

Spearman’s rank correlation coefficient was utilized to evaluate the interrelationship between each feature, to ensure the reliability of the features. Features with a correlation coefficient exceeding 0.9 between any two features were preserved. To enhance the feature representation, a greedy recursive deletion approach was employed to filter the features. This approach involved iteratively eliminating the feature with the highest redundancy in the current set. Subsequently, the least absolute shrinkage and selection operator (LASSO) regression model was utilized to identify the features with nonzero coefficients using the 10-fold cross-validation method. All feature selection procedures were executed in the training cohort and subsequently applied to the test cohort. The LASSO regression modeling was conducted using the Python scikit-learn package.

Features exhibiting nonzero coefficients were retained for fitting the regression model and amalgamated into a radiomics signature. Each patient was then assigned a radiomics score by weighting them with the linear combination of the retained features and their corresponding model coefficients.

### Construction of radiomics models

Various machine learning algorithms were utilized to develop classification models for the optimal identification of insulinomas and NF-PNETs. Following the application of LASSO feature filtering, the selected intratumoral ROI radiomics features were inputted into commonly employed machine learning models such as logistic regression (LR), random forest (RF), extreme gradient boosting (XGBoost), light gradient boosting machine (LightGBM), extra tree, and multilayer perceptron (MLP) models to construct intratumoral radiomics models. The diagnostic effectiveness of various machine learning models was evaluated by assessing metrics including the receiver operator characteristic curve (ROC), area under the curve (AUC), accuracy, specificity, sensitivity, positive predictive value (PPV), and negative predictive value (NPV). Ultimately, the most optimal intratumoral radiomics model was determined, and the selected machine learning algorithm, which demonstrated satisfactory performance, was applied to establish peritumoral and combined radiomics models.

### Radiomics model assessment

An intratumoral radiomics model, peritumoral radiomics model, and combined radiomics model were constructed using a consistent machine learning algorithm. The diagnostic effectiveness of these three radiomics models was assessed in both the training and test cohorts through the construction of ROC curves. Furthermore, a Delong test was employed to compare the performance of these radiomics models in terms of the AUC.

The concordance between the predictions made by various radiomics models and the observed outcomes was evaluated utilizing calculating the calibration curve, which compared the predictions of these models with the actual observations. The calibration performance of these three radiomics models was assessed through the construction of calibration curves, while the Hosmer-Lemeshow (H-L) analytical fit was employed to evaluate the calibration ability of these radiomics models. Furthermore, decision curve analysis (DCA) was utilized to assess the clinical usefulness of these predictive models. Finally, the radiomics model with the best performance was certified and defined as the radiomics signature.

### Nomogram establishment and assessment

Finally, A nomogram was developed in the training cohort to assess the incremental predictive value of the integrated radiomics signature alongside retained clinical features intuitively and efficiently. Utilizing logistic regression analysis, the nomogram was constructed by incorporating the radiomics signature with the retained clinical features. The calibration curve was employed to compare the consistency between the nomogram’s predictions and actual observations. Calibration curves were constructed to assess the calibration of the nomogram models using mean absolute error and 1,000 bootstrap samples with the R CalibrationCurves package. The DCA was employed to evaluate the net benefits of the nomogram models at different high-risk thresholds. The predictive accuracy of the nomogram model was further assessed using the clinical impact curve (CIC). Finally, the performance of the nomogram was determined by analyzing the ROC curves and their corresponding AUC values.

### Statistical analysis

**Figure 3:**
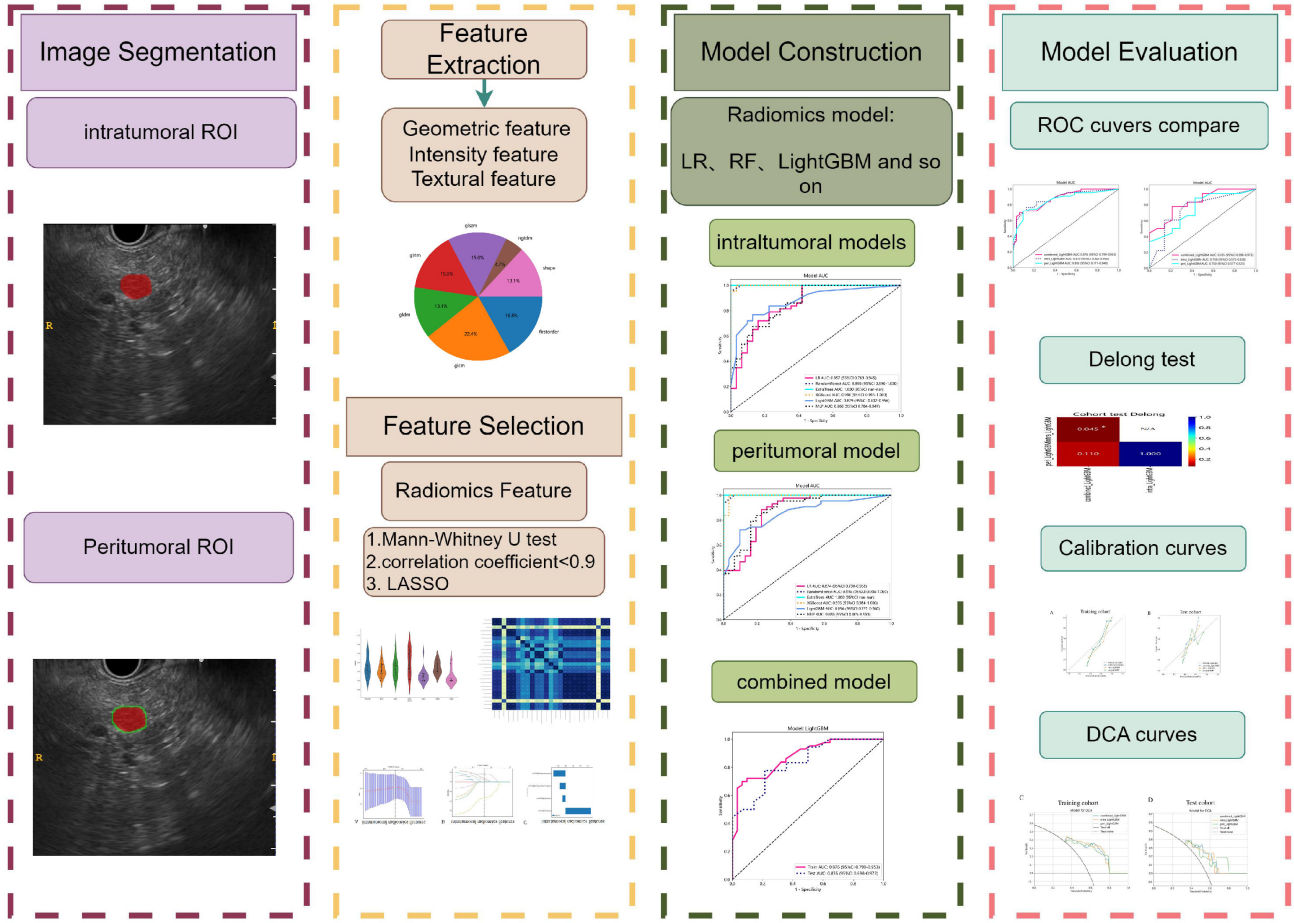
The workflow of this study.

The clinical parameters and radiomics features of the patients were compared using appropriate statistical tests, namely the independent sample t-test, Mann Whitney U test, or X2 test. A threshold of a two-tailed p-value < 0.05 was established to determine statistical significance. The prediction performance of different models was evaluated using metrics such as AUC, accuracy, sensitivity, specificity, PPV, and NPV. The AUC values were compared between any two models using a Delong test to assess their performance. The entire workflow for this study is illustrated in **Figure 3**.

## Results

### Baseline population characteristics

In this retrospective study, a cohort of 106 patients (66 women, 40 men) was enrolled, with 74 patients in the training cohort and 32 patients in the test cohort. In the cohort of patients diagnosed with insulinoma, 19 instances of pancreatic lesions were not identified through contrast-enhanced CT imaging, resulting in a missed diagnosis rate of 31.15% (19 out of 61 cases). Additionally, three cases of NF-PNETs had undetected pancreatic lesions via contrast-enhanced CT. Conversely, EUS successfully detected all pancreatic lesions associated with insulinomas and NF-PNETs in this investigation.

The findings of baseline indicated that there were no significant disparities observed in age, shape, margin characteristics, echo characteristics, uniformity of echo, calcification, location of masses, and the presence of cystic degeneration between patients with insulinomas and NF-PNETs in both the training and test cohorts. Nonetheless, it was noted that insulinomas exhibited a significantly smaller diameter compared to NF-PNETs. The findings suggest that insulinomas closely resemble NF-PNETs and pose challenges in their classification based on macroscopic features observed through EUS, except for diameter. Finally, the diameter was the clinical feature that was ultimately preserved and was utilized to construct the nomogram. **Table 1** and **Supplementary Table 1** provide a comprehensive overview of the clinical and radiological baseline characteristics.

**Table 1:** Clinical and radiological characteristics in the training and test cohorts.

Variable	Training cohort (N=74)			Test cohort (N=32)		
	NF-PNETs	Insulinomas	P-value	NF-PNETs	Insulinomas	P-value
Age	45.29 ± 13.23	43.86 ± 13.18	0.647	54.07 ± 10.90	52.78 ± 11.69	0.751
Maximum diameter	33.88 ± 14.48	13.72 ± 5.04	<0.001	28.68 ± 10.92	13.74 ± 6.85	<0.001
Gender			1.000			0.002
0	18(58.06)	26(60.47)		5(35.71)	17(94.44)	
1	13(41.94)	17(39.53)		9(64.29)	1(5.56)	
Shape			0.609			0.216
0	12(38.71)	13(30.23)		6(42.86)	3(16.67)	
1	19(61.29)	30(69.77)		8(57.14)	15(83.33)	
Margin			0.648			0.819
0	4(12.90)	3(6.98)		2(14.29)	1(5.56)	
1	27(87.10)	40(93.02)		12(85.71)	17(94.44)	
Echo			0.163			1.000
0	2(6.45)	9(20.93)		2(14.29)	2(11.11)	
1	29(93.55)	34(79.07)		12(85.71)	16(88.89)	
Uniformity			0.105			0.267
0	21(67.74)	21(48.84)		6(42.86)	4(22.22)	
1	10(32.26)	22(51.16)		8(57.14)	14(77.78)	
Calcification			0.869			1.000
0	30(96.77)	43(100.00)		14(100.00)	18(100.00)	
1	1(3.23)	0(0.00)		0(0.00)	0(0.00)	
Cystic areas			0.086			1.000
0	26(83.87)	42(97.67)		14(100.00)	18(100.00)	
1	5(16.13)	1(2.33)		0(0.00)	0(0.00)	
Location			0.319			0.556
0	16(51.61)	16(37.21)		7(50.00)	6(33.33)	
1	15(48.39)	27(62.79)		7(50.00)	12(66.67)	

Gender: “0” means female, “1” means male; Shape: “0” means irregular shape, “1” means regular shape; Margin: “0” means unclear margin of lesion, “1” means clear margin of lesion; Echo: “0” means means not hypoechoic of lesion, “1” means hypoechoic of lesion; uniformity: “0” means nonuniformity of echo; “1” means uniformity of echo; Calcification: “0” means no calcification, “1” means calcification; Cystic areas: “0” means no cystic areas, “1” means cystic areas; Location: “0” means head and uncinate process of the pancreas, “1” means body and tail of the pancreas.

### Radiomics feature extraction and screening

**Figure 4:**
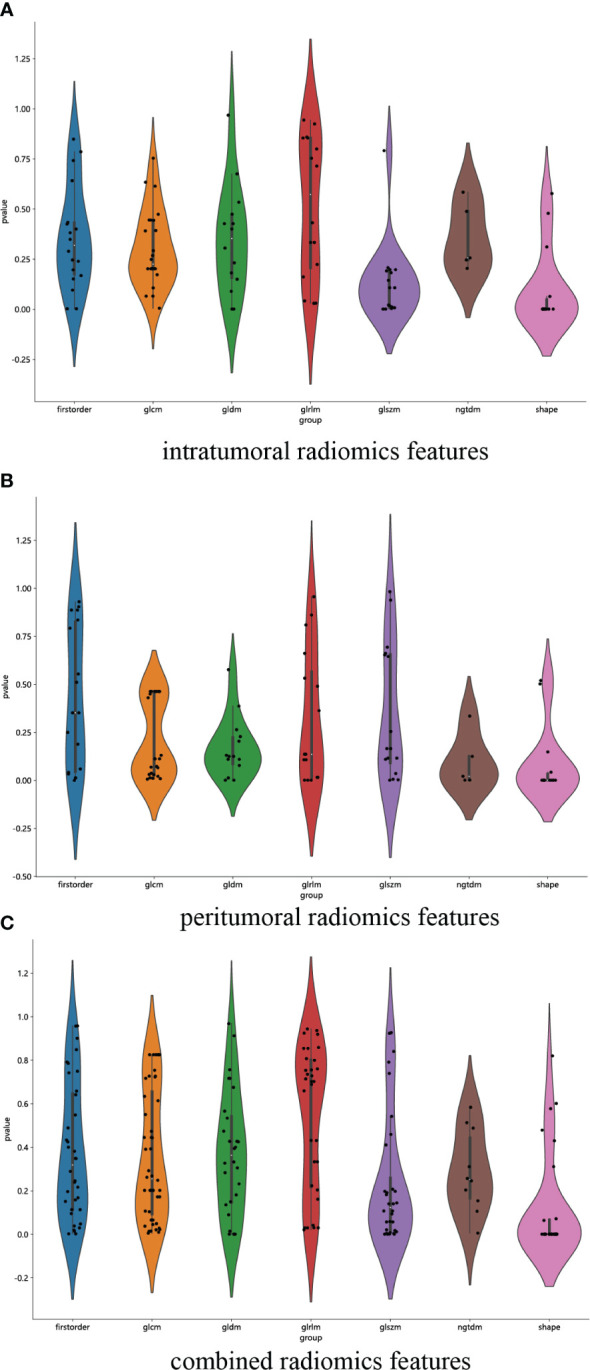
Violin plot for differential analyses of intratumoral **(A)**, peritumoral **(B)**, and combined **(C)** radiomics features with their corresponding *p* values.

**Figure 5:**
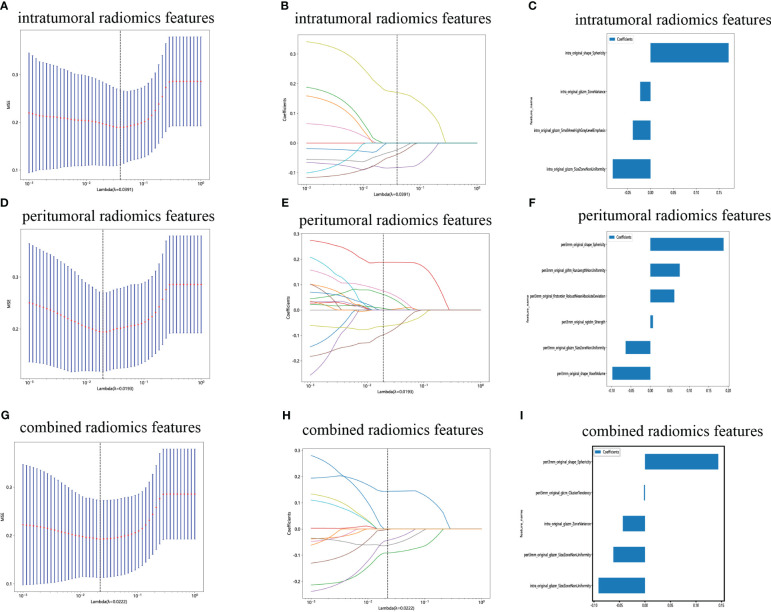
Radiomics feature selection with the LASSO regression model. **(A)** The LASSO model’s tuning parameter (λ) was selected using 10-fold cross-validation via the minimum criterion. The vertical lines illustrate the optimal value of the LASSO tuning parameter (λ) for the intratumoral radiomics features. **(B)** A LASSO coefficient profile plot with different log(λ) values is displayed. The vertical dashed lines represent 9 intratumoral radiomics features with nonzero coefficients selected with the optimal λ value. **(C)** The bar graph of intratumoral radiomics features with their nonzero coefficients. **(D–F)** The same workflow was used for peritumoral radiomics feature analysis. **(G–I)** The same workflow was used for the combined radiomics features analysis. (“intra” means “intratumoral”; “peri3 mm” means “peritumoral region with dilation of 3 mm”).

We have successfully acquired a comprehensive collection of seven categories and 107 radiomics features that were manually derived. These features consist of 18 first-order features, 14 shape features, and the remaining texture features. The specific definitions for these handcrafted features have been previously documented in articles (38). All the comprehensive series of intratumoral radiomics features (**Figure 4A**), peritumoral radiomics features (**Figure 4B**), and combined radiomics features (**Figure 4C**), along with their corresponding *p* values, are displayed in **Figure 4**. A total of four intratumoral radiomics features with nonzero coefficients were retained through the process of feature downsizing and LASSO logistic regression. The coefficients and mean standard errors (MSEs) resulting from the 10-fold validation are presented in **Figures 5A, B**, while the retained intratumoral radiomics features and their coefficients are displayed in **Figure 5C**. Similarly, six peritumoral radiomics features (**Figures 5D–F**) and five combined radiomics features (**Figures 5G–I**) with nonzero coefficients were preserved and exhibited individually.

### Intratumoral radiomics models and performance

**Figure 6:**
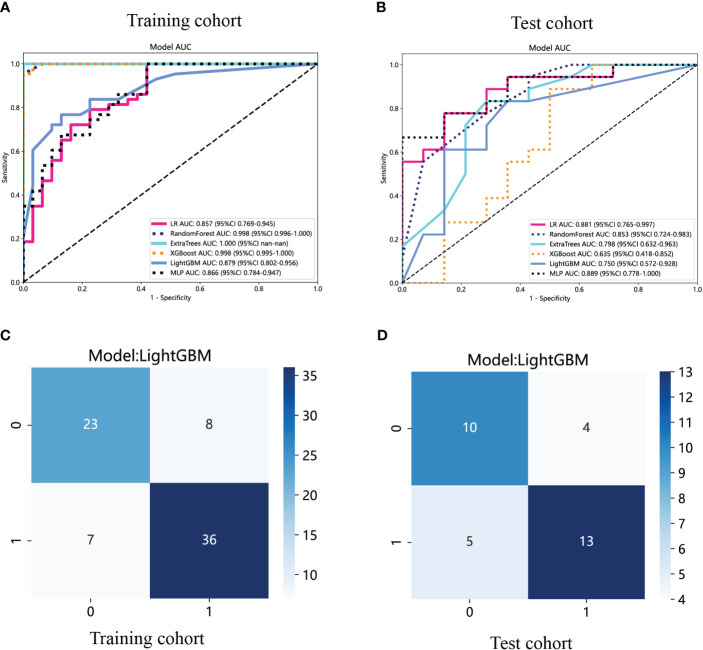
The ROC curves of different intratumoral radiomics models based on six machine learning algorithms for predicting NF-PNETs and insulinomas. **(A)** The ROC curves of different intratumoral radiomics models in the training cohort. **(B)** The ROC curves of different intratumoral radiomics models in the test cohort. **(C)** The confusion matrix of the LightGBM-based intratumoral radiomics model in the training cohort. **(D)** The confusion matrix of the LightGBM-based intratumoral radiomics model in the test cohort.

The ROC curves and AUCs of the six intratumoral radiomics models, generated using the six widely used machine learning algorithms, are depicted in **Figures 6A, B** for the training and test cohorts. Moreover, comprehensive information can be found in **Table 2**. Notably, the RF, ExtraTrees, and XGBoost models exhibited a clear inclination toward overfitting. Additionally, it is important to highlight that the AUCs of the LR and MLP models in the test cohort surpassing those in the training cohort are both inappropriate and lack objectivity. In contrast, the LightGBM model demonstrated superior performance and exhibited stronger consistency between the training (AUC=0.879, 95% CI 0.8019 - 0.9558) and test (AUC=0.750, 95% CI 0.5718 - 0.9282) cohorts. Furthermore, in the training cohort, the LightGBM model outperformed the LR and MLP models, establishing itself as the most effective radiomics model. The LightGBM model demonstrated an accuracy of 0.719, sensitivity of 0.722, specificity of 0.714, PPV of 0.765, and NPV of 0.667 in the test cohort (**Table 2**). Consequently, the LightGBM model was deemed the most appropriate for subsequent analyses and was chosen as the foundational model for constructing intratumoral, peritumoral, and combined radiomics models. The prediction accuracy of the LightGBM model was further visualized through a confusion matrix (**Figures 6C, D**).

**Table 2:** Diagnostic performance of different models for predicting F-PNETs in training and test cohorts.

Model	Cohort	AUC(95% CI)	Accuracy	Sensitivity	Specificity	PPV	NPV
Intratumoral model (LR)	Training	0.857(0.7688 - 0.9447)	0.811	0.977	0.581	0.764	0.947
	Test	0.881(0.7646 - 0.9973)	0.781	0.722	0.857	0.867	0.706
Intratumoral model (RF)	Training	0.998(0.9956 - 1.0000)	0.905	0.837	1.000	1.000	0.816
	Test	0.853(0.7237 - 0.9826)	0.750	0.889	0.571	0.727	0.800
Intratumoral model (ExtraTrees)	Training	1.000(1.0000 - 1.0000)	0.419	0.000	1.000	0.000	0.419
	Test	0.798(0.6320- 0.9633)	0.750	0.722	0.786	0.812	0.687
Intratumoral model (XGBoost)	Training	0.998(0.9949 - 1.0000)	0.973	0.977	0.968	0.977	0.968
	Test	0.635(0.4181 - 0.8517)	0.688	0.833	0.500	0.682	0.700
Intratumoral model (MLP)	Training	0.866(0.7842 - 0.9473)	0.811	0.977	0.581	0.764	0.947
	Test	0.889(0.7776 - 1.0000)	0.781	0.611	1.000	1.000	0.667
Intratumoral model (LightGBM*)	Training	0.879(0.8019 - 0.9558)	0.797	0.744	0.871	0.889	0.711
	Test	0.750(0.5718 - 0.9282)	0.719	0.722	0.714	0.765	0.667
Peritumoral model (LightGBM*)	Training	0.856(0.7805 - 0.9024)	0.770	0.674	0.903	0.906	0.667
	Test	0.750(0.5768 - 0.9232)	0.625	0.667	0.571	0.667	0.571
Combined model (LightGBM*)	Training	0.876(0.7990 - 0.9527)	0.784	0.674	0.935	0.935	0.674
	Test	0.835(0.6978 - 0.9729)	0.688	0.611	0.786	0.786	0.611

*Represents models were constructed based on LightGBM.

LR, logistic regression; RF, random forest; LightGBM, light gradient boosting machine; MLP, multilayer perceptron; XGBoost, extreme gradient boosting; CI, credibility interval.

### Construction and assessment of the peritumoral and combined radiomics models

**Figure 7:**
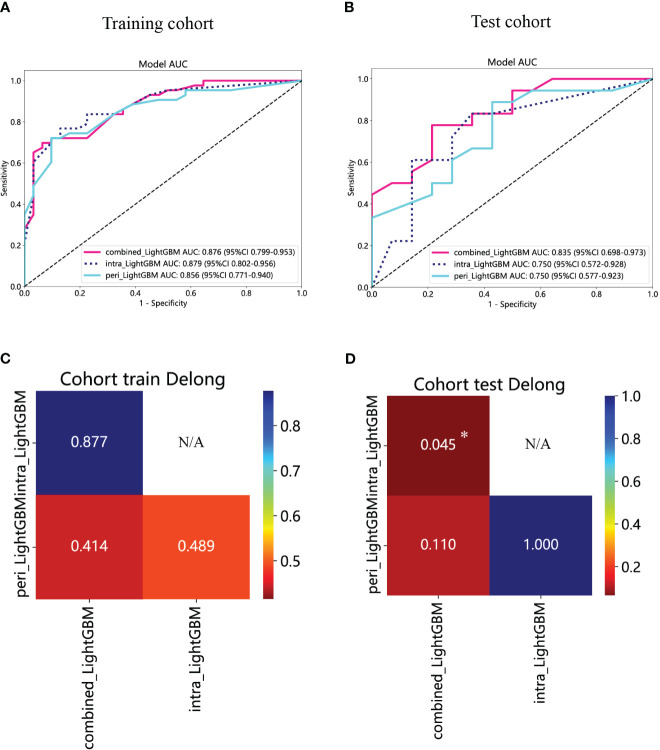
The ROC curves of the intratumoral radiomics model based on LightGBM (abbreviated “intra_ LightGBM”), the peritumoral radiomics model based on LightGBM (abbreviated “peri_ LightGBM”), and the combined radiomics model based on LightGBM (abbreviated “intra- and peri_ LightGBM”) in the training **(A)** and test **(B)** cohorts. The results of the Delong test in the training **(C)** and test **(D)** cohorts. (* indicates *P* < 0.05).

**Figure 8:**
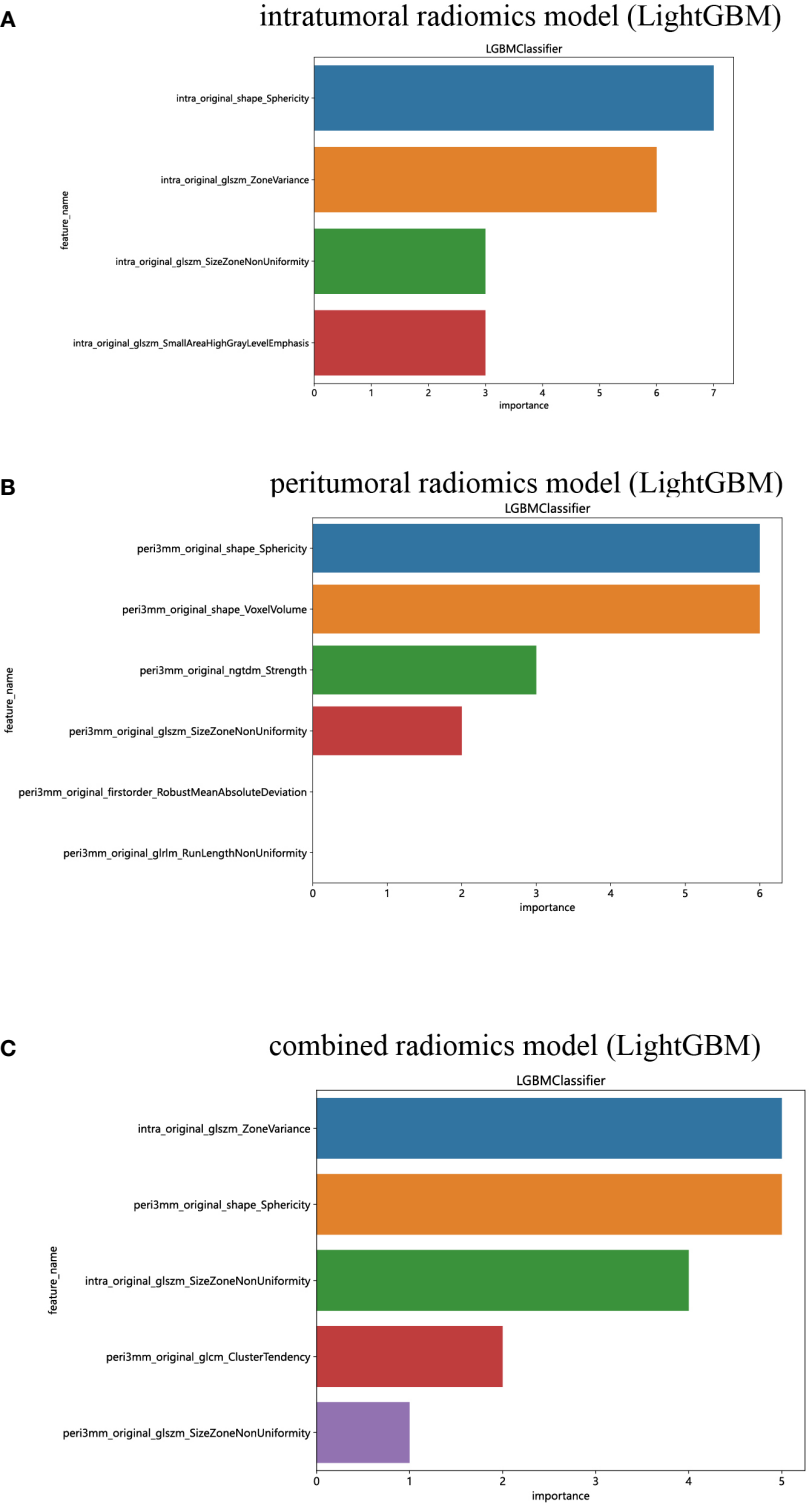
The weight bars graph of the retained radiomics features in intratumoral **(A)**, peritumoral **(B)**, and combined **(C)** radiomics models.

The performance of the peritumoral and combined radiomics LightGBM models in predicting outcomes is presented in **Table 2** for both the training and test cohorts. **Figure 7** displays the ROC curves for the intratumoral radiomics model, peritumoral radiomics model, and combined radiomics model in both the training (**Figure 7A**) and test (**Figure 7B**) cohorts. Among the various models examined, the combined radiomics model exhibited a performance of AUC=0.876 (95% CI 0.7990 - 0.9527) in the training cohort, which was consistent with both the intratumoral and peritumoral models. However, it was observed that the combined radiomics model achieved the highest level of performance in the test cohort (AUC=0.835, 95% CI 0.6978 - 0.9729). Moreover, to objectively evaluate the efficacy of these models, the Delong test was employed. In the training cohort, no statistically significant difference in the AUC was observed among these three models (**Figure 7C**). Furthermore, the AUC of the peritumoral radiomics model was found to be comparable to that of the intratumoral radiomics model (peritumoral model vs. intratumoral model: AUC=0.750 vs. 0.750, p=1.000) (**Table 2**, **Figure 7D**) within the test cohort. This indicates that the peritumoral model’s effectiveness is not inferior to that of the intratumoral model. In contrast, the AUC of the combined model demonstrated a statistically significant increase compared to that of the intratumoral models in the test cohort (combined model vs. intratumoral model: AUC=0.835 vs. 0.750, *p*=0.045) (**Table 2**, **Figure 7D**). This finding suggests that incorporating both intratumoral and peritumoral features may enhance diagnostic effectiveness. **Figure 8** displays the weight bars graph illustrating the retained radiomics features in the intratumoral, peritumoral, and combined radiomics models.

**Figure 9:**
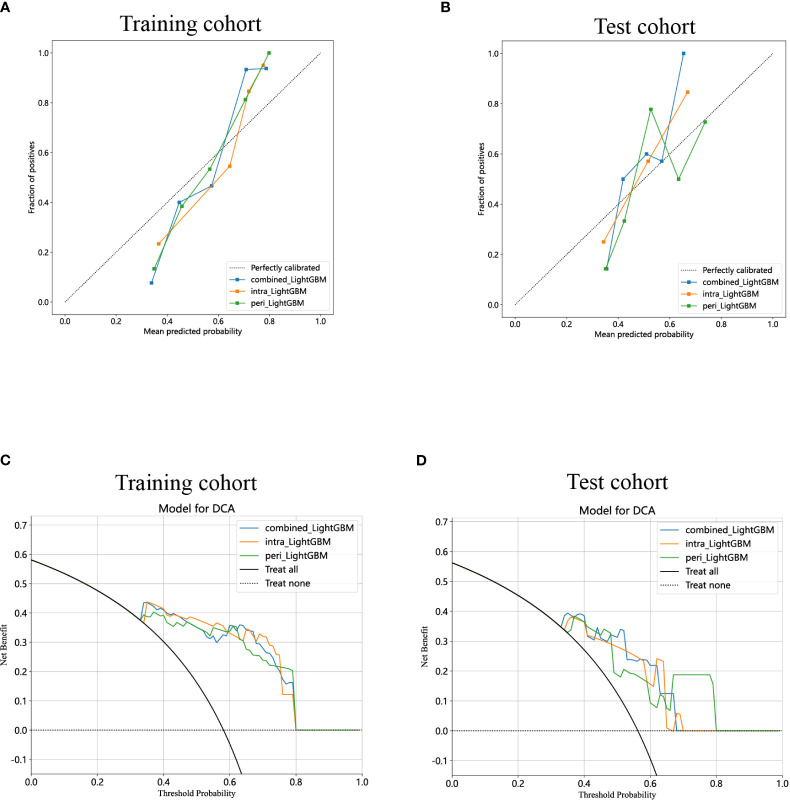
Calibration curves for the intratumoral radiomics model based on LightGBM (abbreviated “intra_ LightGBM”), peritumoral radiomics model based on LightGBM (abbreviated “peri_ LightGBM”), and combined radiomics model (abbreviated “combined_ LightGBM”) in the training **(A)** and test **(B)** cohorts. The DCA curves for the intratumoral, peritumoral, and combined radiomics models based on LightGBM in the training **(C)** and test **(D)** cohorts.

The calibration curves of the combined model exhibited consistency between the predicted and observed insulinomas in both the training and test cohorts. The results of the H-L test demonstrated that all the intratumoral model, peritumoral model, and combined model possessed superior predictive accuracy (**Table 3**). The calibration curves for the training and test cohorts are presented in **Figures 9A, B**, respectively.

**Table 3:** The results of Hosmer-Lemeshow test.

Model	*P*-value	
	Training cohort	Test cohort
Intratumoral radiomics model (LightGBM)	0.519	0.258
Peritumoral radiomics model (LightGBM)	0.553	0.095
Combined radiomics model (LightGBM)	0.170	0.149

**Figure 10:**
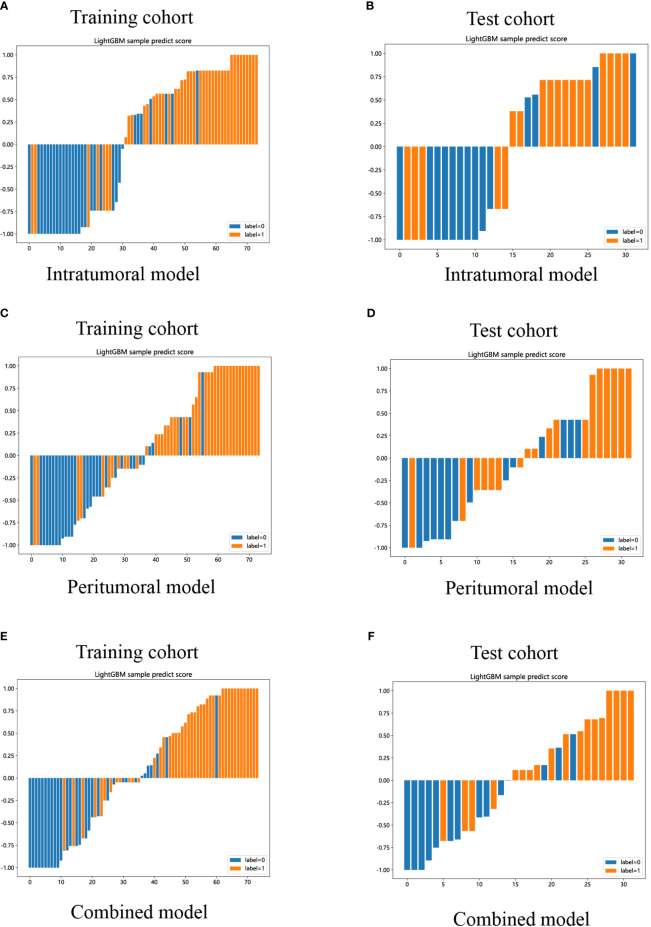
LightGBM-based prediction scores of the intratumoral **(A, B)**, peritumoral **(C, D)**, and combined **(E, F)** radiomics models in the training and test cohorts. (“label=0” means “NF-PNETs”; “label=1” means “insulinomas”).

Lastly, DCA was performed to evaluate the performance of each model, and the findings are depicted in **Figures 9C, D**. The combined model exhibited a remarkable net benefit for patient intervention, as indicated by its prediction probability, in comparison to hypothetical scenarios where no prediction model was available, such as the treat-all or treat-none approaches. Additionally, the combined model consistently demonstrated values similar to those of other models in both the training and test cohorts. Consequently, these three radiomics models hold promise in enhancing the clinical efficacy of predicting insulinomas before pathological examination. The prediction scores of the intratumoral, peritumoral, and combined models are shown in **Figure 10**. Ultimately, the combined radiomics model was validated as the radiomics signature and utilized in the creation of a nomogram, based on its superior performance.

### Nomogram construction and assessment

**Figure 11:**
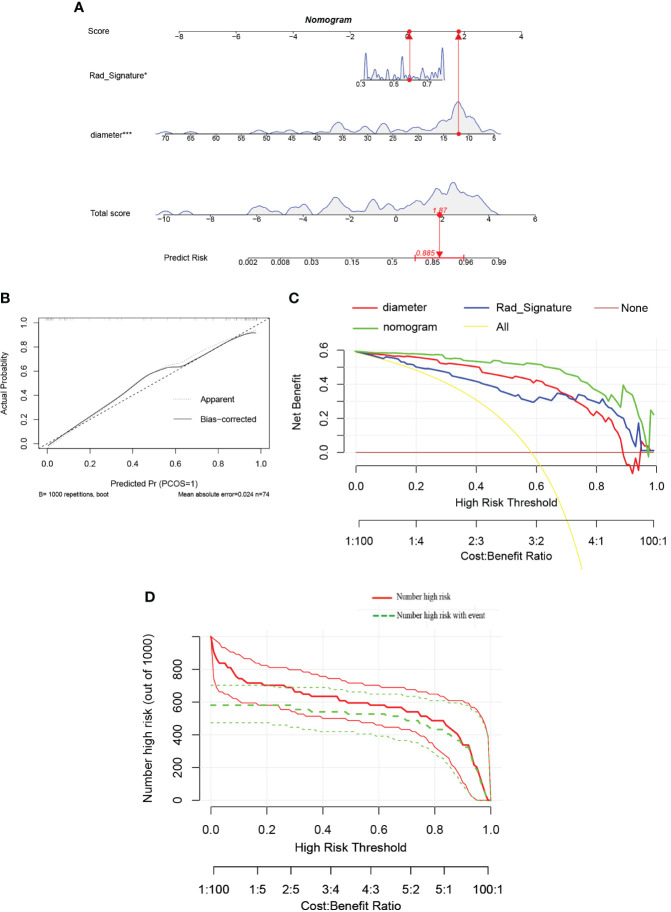
**(A)** The nomogram predicting insulinomas based on diameter and radiomics signature. The nomogram is used by summing all points identified on the scale for each variable. The total points projected on the bottom scales indicate the probabilities of insulinomas. (“Rad_Signature” means “radiomics signature”). **(B)** The calibration curves for the nomogram with the mean absolute error = 0.024. **(C)** Decision curve analysis (DCA) of the nomogram and each strategy (the “All” means diagnosis-all strategy; the”None” means diagnosis-none strategy). **(D)** The clinical impact curve (CIC) of the nomogram.

Additionally, a nomogram was developed using logistic regression analysis of radiomics signature and diameter through the R rms package (**Figure 11A**). Subsequently, a calibration curve was employed to assess the predictive efficacy of the nomogram model. The calibration curve demonstrated minimal error between the actual and predicted probabilities of insulinomas, with a mean absolute error of 0.024, indicating the high accuracy of this nomogram model in predicting insulinomas (**Figure 11B**). The findings of the DCA demonstrated that the “Nomogram” curve exhibited higher values compared to the “All” curve, “diameter” curve, “Rad_Signature” curve, and “None” curve within the high-risk threshold ranging from nearly 0 to 1.0 (**Figure 11C**). It indicated that patients may experience a net benefit from utilizing this nomogram model. Additionally, a CIC was constructed based on the DCA curve to evaluate the clinical efficacy of the nomogram model visually. The proximity of the”Number high risk” curve to the “Number high risk with event” curve at a high-risk threshold ranging from 0.2 to 1.0 suggests that this nomogram model exhibits exceptional predictive capability (**Figure 11D**). These findings further suggested that the diameter of pancreatic mass and radiomics signature may significantly contribute to the prediction of insulinomas.

**Figure 12:**
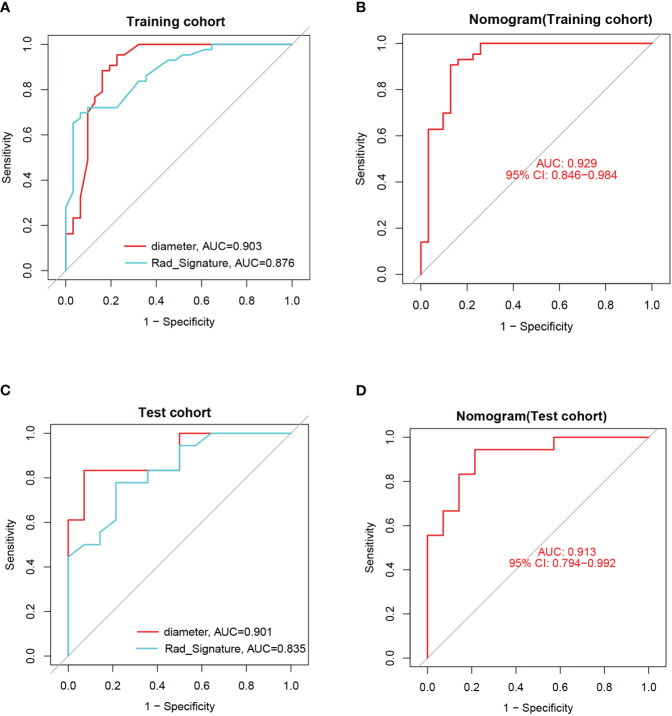
**(A)** The ROCs and AUCs of diameter and radiomics signature for predicting insulinomas in the training cohort. **(B)** The ROC and AUC of the nomogram for predicting insulinomas in the training cohort. **(C)** The ROCs and AUCs of diameter and radiomics signature for predicting insulinomas in the test cohort. **(D)** The ROC and AUC of the nomogram for predicting insulinomas in the test cohort.

ROC curves with AUC were utilized to assess the diagnostic efficacy of this nomogram model in distinguishing insulinomas from NF-PNETs based on the diameter of pancreatic mass and radiomics signature. The analysis of ROC curves indicated AUC values of 0.903 for diameter, 0.876 for radiomics signature, and 0.929 (95% CI, 0.846–0.984) for the nomogram in the training cohort (**Figures 12A, B**). Furthermore, the AUCs for the diameter and nomogram were 0.901 and 0.913(95% CI, 0.794–0.992) in the test cohort, respectively (**Figures 12C, D**).

## Discussion

This study utilized EUS-based radiomics features obtained from intratumoral and peritumoral regions, along with the implementation of six machine learning algorithms, to develop predictive models for discerning insulinomas and NF-PNETs. The results of our investigation demonstrated that the integration of radiomics data from both intratumoral and peritumoral regions yielded the most accurate prediction performance. These findings suggest that peritumoral regions may contain supplementary information that enhances the identification of insulinomas and NF-PNETs. Consistently, prior research has demonstrated that the integration of peritumoral and intratumoral data using a nomogram model, which incorporates deep learning contrast-enhanced ultrasound and clinical characteristics, has exhibited notable proficiency in the identification of preoperative aggressiveness in PNETs (33). Moreover, the effectiveness of employing radiomics, machine learning, and deep learning techniques based on EUS imaging for the prediction of gastrointestinal stromal tumors and pancreatic ductal adenocarcinoma has been substantiated in previous studies (30, 39, 40). However, to the best of our knowledge, we were the first to report on the remarkable predictive capabilities of EUS imaging-based intratumoral and/or peritumoral radiomics models for identifying NF-PNETs and insulinomas.

The spectrum of PNETs encompasses a broad range of biological and clinical characteristics. The majority of PNETs, comprising approximately 80%, are NF-PNETs (41). Furthermore, NF-PNETs, which often originate from the head of the pancreas, demonstrate a higher propensity for aggressiveness due to elevated tumor T-stage, lymph node invasion, and liver metastases. Consequently, patients afflicted with NF-PNETs experience notably inferior overall survival rates compared to their functional counterparts (42). Insulinomas, on the other hand, represent the most prevalent F-PNETs and predominantly manifest as clinically benign, with malignancy observed in only approximately 10% of cases (43, 44). Recurrent hypoglycemia, resulting from abnormal endogenous hyperinsulinism, is a characteristic manifestation of insulinomas. While the excessive secretion of insulin is essential for diagnosing insulinomas, delayed or inaccurate identification of hypoglycemia and other common presentations often result in severe consequences and mortality associated with insulinomas (8). In fact, patients with insulinomas frequently endure misdiagnosis as neurological disorders over extended periods due to the diverse clinical symptoms, nonspecific biochemical tests, and low-specificity clinical prediction models (8, 45). Currently, the 72-hour fasting test is the established diagnostic procedure for insulinomas (12). Nevertheless, many patients decline to undergo this test due to an inability to endure the discomfort associated with hunger, thereby impeding the accurate diagnosis of insulinomas (46). Furthermore, the early-stage differentiation between insulinomas and NF-PNET poses a significant challenge (13). Therefore, it is imperative to investigate innovative approaches for accurately discerning NF-PNETs and insulinomas.

Numerous previous studies have highlighted the heightened sensitivity of EUS in the diagnosis of PNETs and other small lesions within the pancreas, particularly those measuring less than 2cm (47). A meta-analysis encompassing ten prior studies involving a total of 261 participants revealed that EUS exhibited a commendable average predictive accuracy of 90% (with a range of 77-100%) in the diagnosis of PNETs (48). Notably, preoperative EUS imaging for functional PNETs can effectively evaluate the correlation and proximity of the lesion to the main pancreatic duct, thereby playing a crucial role in determining the appropriate surgical approach (49). A considerable number of patients diagnosed with F-PNETs, which frequently occur in conjunction with MEN1, commonly exhibit the presence of multiple small pancreatic lesions. Two-thirds of insulinomas are smaller than 2 cm and 30% are smaller than 1 cm (50). Numerous studies in the literature have demonstrated the superiority of EUS over CT and MRI in detecting small pancreatic lesions (27, 47). Additionally, EUS allows for detailed evaluation of the relationship between lesions and surrounding bile ducts, arteries, and veins before surgery, thereby influencing surgical decision-making (49). Due to the inherent limitations of conventional CT and MRI techniques in effectively detecting these pancreatic minute lesions, the utilization of EUS and contrast-enhanced EUS is highly recommended (28). In our investigation, similar to prior research, certain pancreatic lesions were not detected by contrast-enhanced CT imaging, while EUS demonstrated superior performance. Insulinomas, on the other hand, typically manifest as nonmalignant, solitary tumors that are small in size, measuring less than 2cm (51, 52). Our research findings align with previous studies, as they demonstrate that the average maximum diameter of F-PNETs is less than 2cm, signifying a significant disparity when compared to NF-PNETs.

PNETs are often characterized by low-intensity echoes, well-defined borders, regular round shapes, vascularization, and uniform internal echo patterns (53). Interestingly, our findings also demonstrated that insulinomas and NF-PNETs exhibit comparable features in terms of shape, margin, and uniformity of intertumoral echo in this study, suggesting the challenge of distinguishing between them. Radiomics facilitates the extraction of multidimensional data from medical images, surpassing human visual assessment. The efficacy of predictive models for various tumor types can be enhanced by radiomics, leading to increased reliability and objectivity in diagnosis (54, 55). Particularly noteworthy is a multicenter study that has demonstrated the superior predictive capacity of non-contrast MRI radiomics and combined models in distinguishing Grade 1 and 2/3 NF-PNETs, surpassing the performance of models based on clinical and radiological features (56). Moreover, Gu D’s study demonstrated that radiomic signatures derived from CT imaging had a greater probability of accurately predicting the histologic grading of PNETs (32). Similarly, our findings indicate that the intratumoral radiomics model based on EUS showed effective discrimination between NF-PNETs and F-PNETs, potentially enhancing the use of EUS for diagnosing PNETs.

The current body of radiomics literature on PNETs primarily focuses on the intratumoral regions while neglecting the peritumoral region (56–59). Correspondingly, previous studies have demonstrated the significant predictive capabilities of peritumoral radiomics models about pathological outcomes, lymph node metastasis, and recurrence risk stratification. These findings suggest that the peritumoral region of various tumors, including intrahepatic cholangiocarcinoma, cervical cancer, and breast cancer, may contain additional valuable predictive and diagnostic information (60–62). However, the efficacy of EUS-based peritumoral radiomics methodologies in facilitating the differentiation between NF-PNETs and insulinomas remains uncertain.

From our standpoint, the peritumoral and intratumoral regions may exhibit synergistic effects in discerning NF-PNETs and insulinomas. Therefore, a composite model that incorporates radiomics characteristics from both peritumoral and intratumoral regions was formulated and verified. Ultimately, this combined radiomics model demonstrated consistent performance when compared to intratumoral and peritumoral models in the training cohort, respectively. Interestingly, this combined radiomics model demonstrated the highest area under the curve (AUC=0.835, 95% CI 0.6978-0.9729) in the test cohort, indicating its optimal performance. These results, supported by the DeLong test and H-L test, suggest that the combined radiomics model significantly enhances the predictive efficiency of NF-PNETs and insulinomas. In conclusion, the peritumoral region, particularly the tumor-adjacent parenchyma surrounding the tumor lesions, offers valuable predictive information for NF-PNETs and insulinomas. Regrettably, there is a scarcity of research examining the variances in histological attributes within the peritumoral region between NF-PNETs and insulinomas.

To our best knowledge, our study is the first to document the significant predictive potential of EUS imaging-based intratumoral and/or peritumoral radiomics models for distinguishing between NF-PNETs and F-PNETs, especially insulinomas. These results suggest promising opportunities for improving the predictive capabilities of EUS in predicting NF-PNETs and insulinomas. However, it is important to acknowledge the limitations of this study, including the retrospective nature of the analysis conducted at a single center, which may introduce selection bias. Additionally, the prevalence of insulinomas among the subjects in our study exceeded that of NF-PNETs, a finding that diverges from the conclusions drawn in prior research. Moreover, bias is inherent in the image segmentation procedure as all boundary definitions were derived from manual segmentation (63–65). Specifically, the retrospective analysis with a small sample size, conducted within a single center, may introduce potential selection bias. Therefore, it is imperative for future research on EUS-based radiomics for PNETs to incorporate multiple centers, large sample sizes, prospective designs, and multimodal approaches. Additionally, there is a notable advantage in developing radiomics prediction models based on EUS to accurately predict pathological grading, genetic markers, and epigenetic signatures, such as ATRX/DAXX and ALT, in PNETs (66). Furthermore, the utilization of deep learning techniques and investigation into the underlying biological alterations of peritumor imaging features could effectively address bias and improve the interpretability of the models.

## Conclusion

In summary, a robust radiomics model and nomogram utilizing EUS were developed and verified, integrating the diameter of pancreatic lesions and radiomics characteristics within and surrounding the tumor. These models demonstrated high accuracy in distinguishing NF-PNETs and insulinomas. These findings offer promising prospects for enhancing the clinical utility of EUS in predicting NF-PNETs and insulinomas, thereby providing valuable insights for further research and application in this domain.

## Data availability statement

The original contributions presented in the study are included in the article/**Supplementary Material**. Further inquiries can be directed to the corresponding authors.

## Ethics statement

The studies involving humans were approved by Medical Ethics Committee of The First Affiliated Hospital of Guangxi Medical University (No. 2023-K346-01, 2023-12-29). The studies were conducted in accordance with the local legislation and institutional requirements. The ethics committee/institutional review board waived the requirement of written informed consent for participation from the participants or the participants’ legal guardians/next of kin because this study is a retrospective analysis, does not involve identifiable patient identity information, and has no interference with future diagnosis and treatment of patients.

## Author contributions

SM: Writing – review & editing, Writing – original draft, Visualization, Validation, Supervision, Software, Resources, Project administration, Methodology, Investigation, Funding acquisition, Formal Analysis, Data curation, Conceptualization. CH: Writing – original draft, Software, Methodology, Investigation, Formal analysis, Data curation, Conceptualization. YW: Writing – review & editing, Visualization, Software, Methodology, Investigation, Formal analysis, Data curation. HZ: Writing – review & editing, Software, Investigation, Formal analysis, Data curation, Conceptualization. WW: Writing – review & editing, Visualization, Software, Investigation, Formal Analysis. HJ: Writing – review & editing, Visualization, Validation, Supervision, Software, Resources, Project administration, Methodology, Investigation, Funding acquisition, Formal analysis, Data curation, Conceptualization. SQ: Validation, Supervision, Software, Resources, Project administration, Methodology, Investigation, Funding acquisition, Formal analysis, Data curation, Conceptualization, Writing – review & editing, Writing – original draft, Visualization.

## References

[1] Gaitanidis A Patel D Nilubol N Tirosh A Sadowski S Kebebew E. Markers of systemic inflammatory response are prognostic factors in patients with pancreatic neuroendocrine tumors (PNETs): A prospective analysis. Ann Surg Oncol. (2018) 25:122–30. doi: 10.1245/s10434-017-6241-4 PMC8054768 29134377

[2] Souche R Hobeika C Hain E Gaujoux S. Surgical management of neuroendocrine tumours of the pancreas. J Clin Med. (2020) 9 (9):2993. doi: 10.3390/jcm9092993 32947997 PMC7565036

[3] Fu M Yu L Yang L Chen Y Chen X Hu Q. Predictive value of the preoperative prognostic nutritional index for postoperative progression in patients with pancreatic neuroendocrine neoplasms. Front Nutr. (2022) 9:945833. doi: 10.3389/fnut.2022.945833 36159473 PMC9493178

[4] Capodanno Y Chen Y Schrader J Tomosugi M Sumi S Yokoyama A. Cross-talk among MEN1, p53 and Notch regulates the proliferation of pancreatic neuroendocrine tumor cells by modulating INSM1 expression and subcellular localization. Neoplasia (New York N.Y.). (2021) 23:979–92. doi: 10.1016/j.neo.2021.07.008 PMC8350333 34352404

[5] Chang TM Chu PY Lin HY Huang KW Hung WC Shan YS. PTEN regulates invasiveness in pancreatic neuroendocrine tumors through DUSP19-mediated VEGFR3 dephosphorylation. J Biomed Sci. (2022) 29:92. doi: 10.1186/s12929-022-00875-2 36336681 PMC9639322

[6] Megdanova-Chipeva VG Lamarca A Backen A McNamara MG Barriuso J Sergieva S. Systemic treatment selection for patients with advanced pancreatic neuroendocrine tumours (PanNETs). Cancers. (2020) 12 (7):1988. doi: 10.3390/cancers12071988 32708210 PMC7409353

[7] Lee DW Kim MK Kim HG. Diagnosis of pancreatic neuroendocrine tumors. Clin endoscopy. (2017) 50:537–45. doi: 10.5946/ce.2017.131 PMC5719919 29207856

[8] Mo S Wang Y Wu W Zhao H Jiang H Qin S. Identifying target ion channel-related genes to construct a diagnosis model for insulinoma. Front Genet. (2023) 14:1181307. doi: 10.3389/fgene.2023.1181307 37772258 PMC10523017

[9] Imamura M. Recent standardization of treatment strategy for pancreatic neuroendocrine tumors. World J Gastroenterol. (2010) 16:4519–25. doi: 10.3748/wjg.v16.i36.4519 PMC2945482 20857521

[10] Matsumoto K Watanabe M Takao K Takahashi H Daido H Shibata T. Unmasked insulinoma occasioned by severe hypoglycemic coma immediately postpartum: a case report. BMC endocrine Disord. (2023) 23:168. doi: 10.1186/s12902-023-01415-1 PMC10413590 37563593

[11] Giannis D Moris D Karachaliou GS Tsilimigras DI Karaolanis G Papalampros A. Insulinomas: from diagnosis to treatment. A review of the literature. J B.U.ON.: Off J Balkan Union Oncol. (2020) 25 (3):1302–14. 32862570

[12] Hofland J Falconi M Christ E Castaño JP Faggiano A Lamarca A. European Neuroendocrine Tumor Society 2023 guidance paper for functioning pancreatic neuroendocrine tumour syndromes. J Neuroendocrinol. (2023) 35:e13318. doi: 10.1111/jne.13318 37578384

[13] Karakose E Wang H Inabnet W Thakker RV Libutti S Fernandez-Ranvier G. Aberrant methylation underlies insulin gene expression in human insulinoma. Nat Commun. (2020) 11:5210. doi: 10.1038/s41467-020-18839-1 33060578 PMC7566641

[14] Tong Z Wang L Shi W Zeng Y Zhang H Liu L. Clonal evolution dynamics in primary and metastatic lesions of pancreatic neuroendocrine neoplasms. Front Med. (2021) 8:620988. doi: 10.3389/fmed.2021.620988 PMC8131504 34026777

[15] Si Y Huang C Yuan J Zhang X He Q Lin Z. Analysis of prognostic risk factors of endoscopic submucosal dissection (ESD) and curative resection of gastrointestinal neuroendocrine neoplasms. Contrast media Mol Imaging. (2022) 2022:5248256. doi: 10.1155/2022/5248256 35854772 PMC9286938

[16] Frost M Lines KE Thakker RV. Current and emerging therapies for PNETs in patients with or without MEN1. Nat Rev Endocrinol. (2018) 14:216–27. doi: 10.1038/nrendo.2018.3 PMC6538535 29449689

[17] Partelli S Massironi S Zerbi A Niccoli P Kwon W Landoni L. Management of asymptomatic sporadic non-functioning pancreatic neuroendocrine neoplasms no larger than 2 cm: interim analysis of prospective ASPEN trial. Br J Surg. (2022) 109:1186–90. doi: 10.1093/bjs/znac267 PMC10364756 35986682

[18] Partelli S Ramage JK Massironi S Zerbi A Kim HB Niccoli P. Management of asymptomatic sporadic nonfunctioning pancreatic neuroendocrine neoplasms (ASPEN) ≤2 cm: study protocol for a prospective observational study. Front Med. (2020) 7:598438. doi: 10.3389/fmed.2020.598438 PMC7785972 33425946

[19] Plas P Limana L Carré D Thiongane A Raguin O Mansi R. Comparison of the anti-tumour activity of the somatostatin receptor (SST) antagonist [(177)Lu]Lu-satoreotide tetraxetan and the agonist [(177)Lu]Lu-DOTA-TATE in mice bearing AR42J SST(2)-positive tumours. Pharm (Basel Switzerland). (2022) 15 (9):1085. doi: 10.3390/ph15091085 PMC9506113 36145306

[20] Liu T Xu Q Zou X Zhu L Zhao Y. Mind the tributary of the canal: Are stents necessary for insulinoma enucleation in proximity to a prominent Duct of Santorini: A case report and literature review. Medicine. (2022) 101:e31211. doi: 10.1097/MD.0000000000031211 36316943 PMC9622601

[21] Wang H Lin Z Li G Zhang D Yu D Lin Q. Validation and modification of staging Systems for Poorly Differentiated Pancreatic Neuroendocrine Carcinoma. BMC Cancer. (2020) 20:188. doi: 10.1186/s12885-020-6634-9 32138704 PMC7059325

[22] Saleh M Bhosale PR Yano M Itani M Elsayes AK Halperin D. New frontiers in imaging including radiomics updates for pancreatic neuroendocrine neoplasms. Abdominal Radiol (New York). (2022) 47:3078–100. doi: 10.1007/s00261-020-02833-8 33095312

[23] Morgan AD Ramai D Bandaru P Crino SF Facciorusso A. Endoscopic ultrasound-guided therapies in patients with pancreatic neuroendocrine tumors. Endocrine Metab Immune Disord Drug Targets. (2023) 23:1355–8. doi: 10.2174/1871530323666230411141412 37055906

[24] Zhou W Fang Y Han X Kuang T Xu X Lou W. The value of alkaline phosphatase-to-albumin ratio in detecting synchronous metastases and predicting postoperative relapses among patients with well-differentiated pancreatic neuroendocrine neoplasms. J Oncol. (2020) 2020:8927531. doi: 10.1155/2020/8927531 32089687 PMC7026734

[25] Câmara-de-Souza AB Toyoshima MTK Giannella ML Freire DS Camacho CP Lourenço DM Jr. Insulinoma: A retrospective study analyzing the differences between benign and Malignant tumors. Pancreatology: Off J Int Assoc Pancreatology (IAP). (2018) 18:298–303. doi: 10.1016/j.pan.2018.01.009 29452754

[26] Zakaria A Hammad N Vakhariya C Raphael M. Somatostatinoma presented as double-duct sign. Case Rep gastrointestinal Med. (2019) 2019:9506405. doi: 10.1155/2019/9506405 PMC6532322 31210994

[27] Melita G Pallio S Tortora A Crinò SF Macrì A Dionigi G. Diagnostic and interventional role of endoscopic ultrasonography for the management of pancreatic neuroendocrine neoplasms. J Clin Med. (2021) 10 (12):2638. doi: 10.3390/jcm10122638 34203922 PMC8232656

[28] Costache MI Cazacu IM Dietrich CF Petrone MC Arcidiacono PG Giovannini M. Clinical impact of strain histogram EUS elastography and contrast-enhanced EUS for the differential diagnosis of focal pancreatic masses: A prospective multicentric study. Endoscopic ultrasound. (2020) 9:116–21. doi: 10.4103/eus.eus_69_19 PMC7279079 32295969

[29] Tong P Sun D Chen G Ni J Li Y. Biparametric magnetic resonance imaging-based radiomics features for prediction of lymphovascular invasion in rectal cancer. BMC Cancer. (2023) 23:61. doi: 10.1186/s12885-023-10534-w 36650498 PMC9847040

[30] Parasher G Wong M Rawat M. Evolving role of artificial intelligence in gastrointestinal endoscopy. World J Gastroenterol. (2020) 26:7287–98. doi: 10.3748/wjg.v26.i46.7287 PMC7739161 33362384

[31] Zhu HB Zhu HT Jiang L Nie P Hu J Tang W. Radiomics analysis from magnetic resonance imaging in predicting the grade of nonfunctioning pancreatic neuroendocrine tumors: a multicenter study. Eur Radiol. (2023) 34 (1):90–102. doi: 10.1007/s00330-023-09957-7 PMC10791720 37552258

[32] Mori M Palumbo D Muffatti F Partelli S Mushtaq J Andreasi V. Prediction of the characteristics of aggressiveness of pancreatic neuroendocrine neoplasms (PanNENs) based on CT radiomic features. Eur Radiol. (2023) 33:4412–21. doi: 10.1007/s00330-022-09351-9 36547673

[33] Huang J Xie X Wu H Zhang X Zheng Y Xie X. Development and validation of a combined nomogram model based on deep learning contrast-enhanced ultrasound and clinical factors to predict preoperative aggressiveness in pancreatic neuroendocrine neoplasms. Eur Radiol. (2022) 32:7965–75. doi: 10.1007/s00330-022-08703-9 35389050

[34] Du N Shu W Li K Deng Y Xu X Ye Y. An initial study on the predictive value using multiple MRI characteristics for Ki-67 labeling index in glioma. J Trans Med. (2023) 21:119. doi: 10.1186/s12967-023-03950-w PMC9922464 36774480

[35] Zhuo Y Feng M Yang S Zhou L Ge D Lu S. Radiomics nomograms of tumors and peritumoral regions for the preoperative prediction of spread through air spaces in lung adenocarcinoma. Trans Oncol. (2020) 13:100820. doi: 10.1016/j.tranon.2020.100820 PMC7334418 32622312

[36] Pérez-Morales J Tunali I Stringfield O Eschrich SA Balagurunathan Y Gillies RJ. Peritumoral and intratumoral radiomic features predict survival outcomes among patients diagnosed in lung cancer screening. Sci Rep. (2020) 10:10528. doi: 10.1038/s41598-020-67378-8 32601340 PMC7324394

[37] Zwanenburg A Vallières M Abdalah MA Aerts HJWL Andrearczyk V Apte A. The image biomarker standardization initiative: standardized quantitative radiomics for high-throughput image-based phenotyping. Radiology. (2020) 295 (2):328–38. doi: 10.1148/radiol.2020191145 PMC7193906 32154773

[38] Lambin P Leijenaar RTH Deist TM Peerlings J de Jong EEC van Timmeren J. Radiomics: the bridge between medical imaging and personalized medicine. Nat Rev Clin Oncol. (2017) 14:749–62. doi: 10.1038/nrclinonc.2017.141 28975929

[39] Gu J Pan J Hu J Dai L Zhang K Wang B. Prospective assessment of pancreatic ductal adenocarcinoma diagnosis from endoscopic ultrasonography images with the assistance of deep learning. Cancer. (2023) 129:2214–23. doi: 10.1002/cncr.34772 36999572

[40] Zhang XD Zhang L Gong TT Wang ZR Guo KL Li J. A combined radiomic model distinguishing GISTs from leiomyomas and schwannomas in the stomach based on endoscopic ultrasonography images. J Appl Clin Med Phys. (2023) 24:e14023. doi: 10.1002/acm2.14023 37166416 PMC10338752

[41] Pan J Bao Q Enders G. The altered metabolic molecular signatures contribute to the RAD001 resistance in gastric neuroendocrine tumor. Front Oncol. (2020) 10:546. doi: 10.3389/fonc.2020.00546 32373532 PMC7186336

[42] Kos-Kudła B Castaño JP Denecke T Grande E Kjaer A Koumarianou A. European Neuroendocrine Tumour Society (ENETS) 2023 guidance paper for nonfunctioning pancreatic neuroendocrine tumours. J Neuroendocrinol. (2023) 35:e13343. doi: 10.1111/jne.13343 37877341

[43] Munro V McDonell LM Keough V Siddiqi FS. Insulinoma presenting as hypoglycemia during lactose tolerance testing: a case report. J Med Case Rep. (2020) 14:96. doi: 10.1186/s13256-020-02419-4 32605595 PMC7325648

[44] Lou H Guo L. Effect of HIV-1 protease inhibitor on IL-18 and IL-1β in rats with insulinoma. Dis Markers. (2022) 2022:1868749. doi: 10.1155/2022/1868749 35601743 PMC9117038

[45] Aslam S Siddiqi AI Shafiq W Azmat U Irfan H Rafaey W. Insulinoma mimicking psychiatric illness: A covert endocrine tumor. Cureus. (2023) 15 (1):e33788. doi: 10.7759/cureus.33788 36819415 PMC9926943

[46] Liao J Ding F Luo W Nie X He Y Li G. Using the secretion ratios of insulin and C-peptide during the 2-h oral glucose tolerance test to diagnose insulinoma. Digestive Dis Sci. (2021) 66 (5):1533–9. doi: 10.1007/s10620-020-06379-z 32529519

[47] Zilli A Arcidiacono PG Conte D Massironi S. Clinical impact of endoscopic ultrasonography on the management of neuroendocrine tumors: lights and shadows. Digestive liver disease: Off J Ital Soc Gastroenterol Ital Assoc Study Liver. (2018) 50:6–14. doi: 10.1016/j.dld.2017.10.007 29102525

[48] Sundin A Vullierme MP Kaltsas G Plöckinger U. ENETS Consensus Guidelines for the Standards of Care in Neuroendocrine Tumors: radiological examinations. Neuroendocrinology. (2009) 90:167–83. doi: 10.1159/000184855 19077417

[49] Giuliani T Marchegiani G Girgis MD Crinò SF Muthusamy VR Bernardoni L. Endoscopic placement of pancreatic stent for “Deep” pancreatic enucleations operative technique and preliminary experience at two high-volume centers. Surg endoscopy. (2020) 34:2796–802. doi: 10.1007/s00464-020-07501-y 32180000

[50] Hoskovec D Krška Z Škrha J Klobušický P Dytrych P. Diagnosis and surgical management of insulinomas-A 23-year single-center experience. Medicina (Kaunas Lithuania). (2023) 59 (8):1423. doi: 10.3390/medicina59081423 37629713 PMC10456644

[51] Kumar S Melek M Rohl P. Case report: hypoglycemia due to metastatic insulinoma in insulin-dependent type 2 diabetes successfully treated with 177 lu-DOTATATE. Front Endocrinol. (2022) 13:906012. doi: 10.3389/fendo.2022.906012 PMC9171402 35685218

[52] Konukiewitz B Jesinghaus M Kasajima A Klöppel G. Neuroendocrine neoplasms of the pancreas: diagnosis and pitfalls. Virchows Archiv: an Int J Pathol. (2022) 480:247–57. doi: 10.1007/s00428-021-03211-5 PMC8986719 34647171

[53] Di Leo M Poliani L Rahal D Auriemma F Anderloni A Ridolfi C. Pancreatic neuroendocrine tumours: the role of endoscopic ultrasound biopsy in diagnosis and grading based on the WHO 2017 classification. Digestive Dis (Basel Switzerland). (2019) 37:325–33. doi: 10.1159/000499172 30897588

[54] Li X Jiang F Guo Y Jin Z Wang Y. Computer-aided diagnosis of gastrointestinal stromal tumors: a radiomics method on endoscopic ultrasound image. Int J Comput assisted Radiol Surg. (2019) 14:1635–45. doi: 10.1007/s11548-019-01993-3 31049803

[55] Geng X Zhang Y Li Y Cai Y Liu J Geng T. Radiomics-clinical nomogram for preoperative lymph node metastasis prediction in esophageal carcinoma. Br J Radiol. (2024) 97 (1155):652–9. doi: 10.1093/bjr/tqae009 PMC11027331 38268475

[56] Zhu HB Zhu HT Jiang L Nie P Hu J Tang W. Radiomics analysis from magnetic resonance imaging in predicting the grade of nonfunctioning pancreatic neuroendocrine tumors: a multicenter study. Eur Radiol. (2024) 34:90–102. doi: 10.1007/s00330-023-09957-7 37552258 PMC10791720

[57] Bian Y Li J Cao K Fang X Jiang H Ma C. Magnetic resonance imaging radiomic analysis can preoperatively predict G1 and G2/3 grades in patients with NF-pNETs. Abdominal Radiol (New York). (2021) 46:667–80. doi: 10.1007/s00261-020-02706-0 32808056

[58] Benedetti G Mori M Panzeri MM Barbera M Palumbo D Sini C. CT-derived radiomic features to discriminate histologic characteristics of pancreatic neuroendocrine tumors. La Radiologia Med. (2021) 126:745–60. doi: 10.1007/s11547-021-01333-z 33523367

[59] Mori M Benedetti G Partelli S Sini C Andreasi V Broggi S. Ct radiomic features of pancreatic neuroendocrine neoplasms (panNEN) are robust against delineation uncertainty. Physica medica: PM: an Int J devoted to Appl Phys to Med biology: Off J Ital Assoc Biomed Phys (AIFB). (2019) 57:41–6. doi: 10.1016/j.ejmp.2018.12.005 30738530

[60] Xie N Fan X Chen D Chen J Yu H He M. Peritumoral and intratumoral texture features based on multiparametric MRI and multiple machine learning methods to preoperatively evaluate the pathological outcomes of pancreatic cancer. J magnetic resonance imaging: JMRI. (2023) 58:379–91. doi: 10.1002/jmri.28538 36426965

[61] Shi J Dong Y Jiang W Qin F Wang X. MRI-based peritumoral radiomics analysis for preoperative prediction of lymph node metastasis in early-stage cervical cancer: A multi-center study. Magnetic resonance Imaging. (2022) 88:1–8. doi: 10.1016/j.mri.2021.12.008 34968703

[62] Sun Q Lin X Zhao Y Li L Yan K Liang D. Deep learning vs. Radiomics for predicting axillary lymph node metastasis of breast cancer using ultrasound images: don’t forget the peritumoral region. Front Oncol. (2020) 10:53. doi: 10.3389/fonc.2020.00053 32083007 PMC7006026

[63] Lohmann P Bousabarah K Hoevels M Treuer H. Radiomics in radiation oncology-basics, methods, and limitations. Strahlentherapie und Onkologie: Organ der Deutschen Rontgengesellschaft. (2020) 196:848–55. doi: 10.1007/s00066-020-01663-3 PMC7498498 32647917

[64] Franchellucci G Andreozzi M Carrara S De Luca L Auriemma F Paduano D. Contrast enhanced EUS for predicting solid pancreatic neuroendocrine tumor grade and aggressiveness. Diagnostics (Basel Switzerland). (2023) 13 (2):239. doi: 10.3390/diagnostics13020239 PMC9857765 36673049

[65] Tang A Tian L Gao K Liu R Hu S Liu J. Contrast-enhanced harmonic endoscopic ultrasound (CH-EUS) MASTER: A novel deep learning-based system in pancreatic mass diagnosis. Cancer Med. (2023) 12:7962–73. doi: 10.1002/cam4.5578 PMC10134340 36606571

[66] Rindi G Mete O Uccella S Basturk O La Rosa S Brosens LAA. Overview of the 2022 WHO classification of neuroendocrine neoplasms. Endocrine Pathol. (2022) 33:115–54. doi: 10.1007/s12022-022-09708-2 35294740

