# Supplementary material for: Endoscopic ultrasonography-based intratumoral and peritumoral machine learning radiomics analyses for distinguishing insulinomas from non-functional pancreatic neuroendocrine tumors
Source: Front Endocrinol (Lausanne). 2024 Jun 17;15:1383814. doi: 10.3389/fendo.2024.1383814 (PMC11215175; doi:10.3389/fendo.2024.1383814)
Supplement: Supplementary file 1 [file DataSheet_1.pdf]

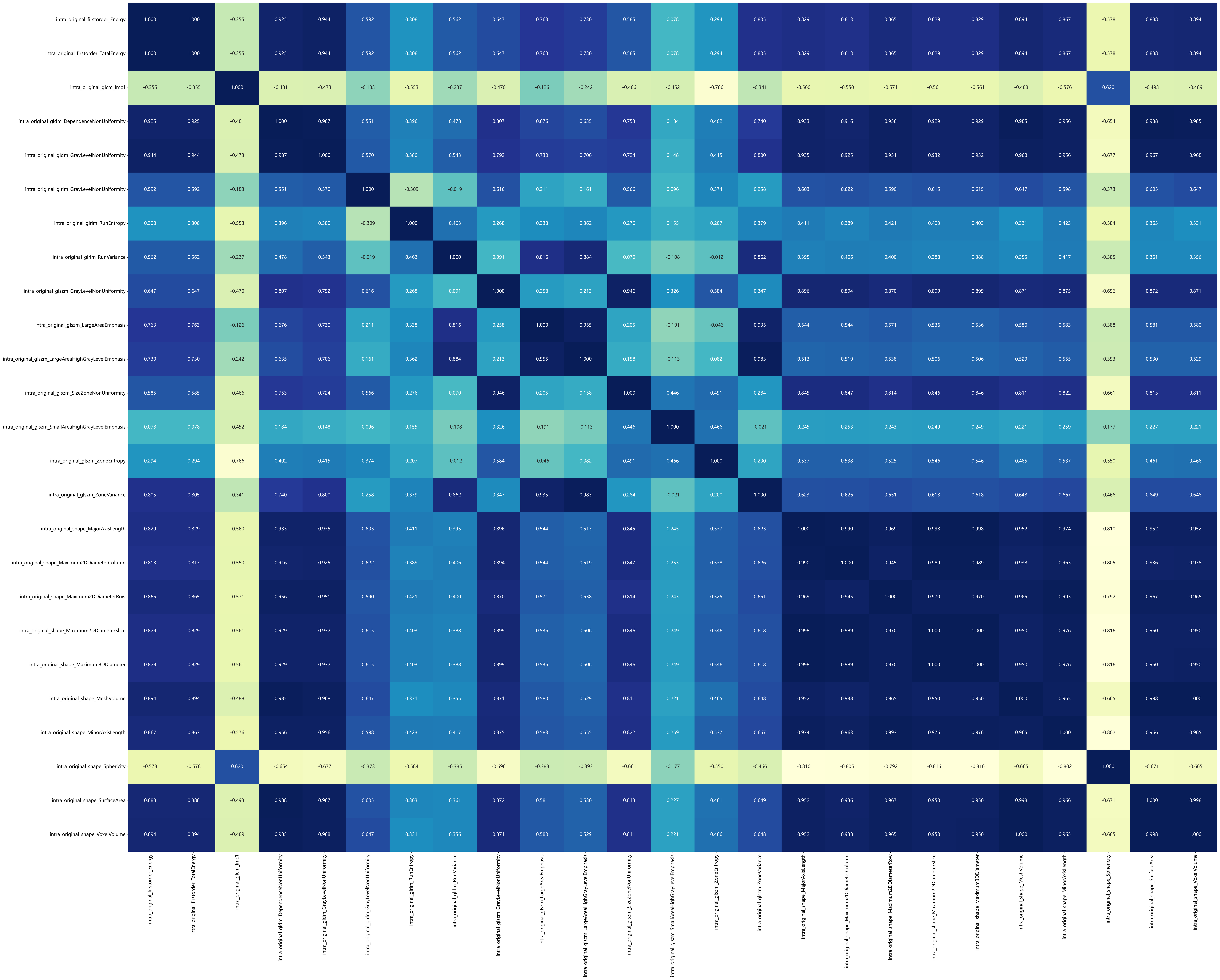
intra_original_firstorder_Energy 1.000 1.000 -0.355 0.925 0.944 0.592 0.308 0.562 0.647 0.763 0.730 0.585 0.078 0.294 0.805 0.829 0.813 0.865 0.829 0.829 0.894 0.867 -0.578 0.888 0.894
intra_original_firstorder_TotalEnergy 1.000 1.000 -0.355 0.925 0.944 0.592 0.308 0.562 0.647 0.763 0.730 0.585 0.078 0.294 0.805 0.829 0.813 0.865 0.829 0.829 0.894 0.867 -0.578 0.888 0.894
intra_original_glcm_Imc1 -0.355 -0.355 1.000 -0.481 -0.473 -0.183 -0.553 -0.237 -0.470 -0.126 -0.242 -0.466 -0.452 -0.766 -0.341 -0.560 -0.550 -0.571 -0.561 -0.561 -0.488 -0.576 0.620 -0.493 -0.489
intra_original_gldm_DependenceNonUniformity 0.925 0.925 -0.481 1.000 0.987 0.551 0.396 0.478 0.807 0.676 0.635 0.753 0.184 0.402 0.740 0.933 0.916 0.956 0.929 0.929 0.985 0.956 -0.654 0.988 0.985
intra_original_gldm_GrayLevelNonUniformity 0.944 0.944 -0.473 0.987 1.000 0.570 0.380 0.543 0.792 0.730 0.706 0.724 0.148 0.415 0.800 0.935 0.925 0.951 0.932 0.932 0.968 0.956 -0.677 0.967 0.968
intra_original_glrlm_GrayLevelNonUniformity 0.592 0.592 -0.183 0.551 0.570 1.000 -0.309 -0.019 0.616 0.211 0.161 0.566 0.096 0.374 0.258 0.603 0.622 0.590 0.615 0.615 0.647 0.598 -0.373 0.605 0.647
intra_original_glrlm_RunEntropy 0.308 0.308 -0.553 0.396 0.380 -0.309 1.000 0.463 0.268 0.338 0.362 0.276 0.155 0.207 0.379 0.411 0.389 0.421 0.403 0.403 0.331 0.423 -0.584 0.363 0.331
intra_original_glrlm_RunVariance 0.562 0.562 -0.237 0.478 0.543 -0.019 0.463 1.000 0.091 0.816 0.884 0.070 -0.108 -0.012 0.862 0.395 0.406 0.400 0.388 0.388 0.355 0.417 -0.385 0.361 0.356
intra_original_glszm_GrayLevelNonUniformity 0.647 0.647 -0.470 0.807 0.792 0.616 0.268 0.091 1.000 0.258 0.213 0.946 0.326 0.584 0.347 0.896 0.894 0.870 0.899 0.899 0.871 0.875 -0.696 0.872 0.871
intra_original_glszm_LargeAreaEmphasis 0.763 0.763 -0.126 0.676 0.730 0.211 0.338 0.816 0.258 1.000 0.955 0.205 -0.191 -0.046 0.935 0.544 0.544 0.571 0.536 0.536 0.580 0.583 -0.388 0.581 0.580
intra_original_glszm_LargeAreaHighGrayLevelEmphasis 0.730 0.730 -0.242 0.635 0.706 0.161 0.362 0.884 0.213 0.955 1.000 0.158 -0.113 0.082 0.983 0.513 0.519 0.538 0.506 0.506 0.529 0.555 -0.393 0.530 0.529
intra_original_glszm_SizeZoneNonUniformity 0.585 0.585 -0.466 0.753 0.724 0.566 0.276 0.070 0.946 0.205 0.158 1.000 0.446 0.491 0.284 0.845 0.847 0.814 0.846 0.846 0.811 0.822 -0.661 0.813 0.811
intra_original_glszm_SmallAreaHighGrayLevelEmphasis 0.078 0.078 -0.452 0.184 0.148 0.096 0.155 -0.108 0.326 -0.191 -0.113 0.446 1.000 0.466 -0.021 0.245 0.253 0.243 0.249 0.249 0.221 0.259 -0.177 0.227 0.221
intra_original_glszm_ZoneEntropy 0.294 0.294 -0.766 0.402 0.415 0.374 0.207 -0.012 0.584 -0.046 0.082 0.491 0.466 1.000 0.200 0.537 0.538 0.525 0.546 0.546 0.465 0.537 -0.550 0.461 0.466
intra_original_glszm_ZoneVariance 0.805 0.805 -0.341 0.740 0.800 0.258 0.379 0.862 0.347 0.935 0.983 0.284 -0.021 0.200 1.000 0.623 0.626 0.651 0.618 0.618 0.648 0.667 -0.466 0.649 0.648
intra_original_shape_MajorAxisLength 0.829 0.829 -0.560 0.933 0.935 0.603 0.411 0.395 0.896 0.544 0.513 0.845 0.245 0.537 0.623 1.000 0.990 0.969 0.998 0.998 0.952 0.974 -0.810 0.952 0.952
intra_original_shape_Maximum2DDiameterColumn 0.813 0.813 -0.550 0.916 0.925 0.622 0.389 0.406 0.894 0.544 0.519 0.847 0.253 0.538 0.626 0.990 1.000 0.945 0.989 0.989 0.938 0.963 -0.805 0.936 0.938
intra_original_shape_Maximum2DDiameterRow 0.865 0.865 -0.571 0.956 0.951 0.590 0.421 0.400 0.870 0.571 0.538 0.814 0.243 0.525 0.651 0.969 0.945 1.000 0.970 0.970 0.965 0.993 -0.792 0.967 0.965
intra_original_shape_Maximum2DDiameterSlice 0.829 0.829 -0.561 0.929 0.932 0.615 0.403 0.388 0.899 0.536 0.506 0.846 0.249 0.546 0.618 0.998 0.989 0.970 1.000 1.000 0.950 0.976 -0.816 0.950 0.950
intra_original_shape_Maximum3DDiameter 0.829 0.829 -0.561 0.929 0.932 0.615 0.403 0.388 0.899 0.536 0.506 0.846 0.249 0.546 0.618 0.998 0.989 0.970 1.000 1.000 0.950 0.976 -0.816 0.950 0.950
intra_original_shape_MeshVolume 0.894 0.894 -0.488 0.985 0.968 0.647 0.331 0.355 0.871 0.580 0.529 0.811 0.221 0.465 0.648 0.952 0.938 0.965 0.950 0.950 1.000 0.965 -0.665 0.998 1.000
intra_original_shape_MinorAxisLength 0.867 0.867 -0.576 0.956 0.956 0.598 0.423 0.417 0.875 0.583 0.555 0.822 0.259 0.537 0.667 0.974 0.963 0.993 0.976 0.976 0.965 1.000 -0.802 0.966 0.965
intra_original_shape_Sphericity -0.578 -0.578 0.620 -0.654 -0.677 -0.373 -0.584 -0.385 -0.696 -0.388 -0.393 -0.661 -0.177 -0.550 -0.466 -0.810 -0.805 -0.792 -0.816 -0.816 -0.665 -0.802 1.000 -0.671 -0.665
intra_original_shape_SurfaceArea 0.888 0.888 -0.493 0.988 0.967 0.605 0.363 0.361 0.872 0.581 0.530 0.813 0.227 0.461 0.649 0.952 0.936 0.967 0.950 0.950 0.998 0.966 -0.671 1.000 0.998
intra_original_shape_VoxelVolume 0.894 0.894 -0.489 0.985 0.968 0.647 0.331 0.356 0.871 0.580 0.529 0.811 0.221 0.466 0.648 0.952 0.938 0.965 0.950 0.950 1.000 0.965 -0.665 0.998 1.000
intra_original_firstorder_Energy
intra_original_firstorder_TotalEnergy
intra_original_glcm_Imc1
intra_original_gldm_DependenceNonUniformity
intra_original_gldm_GrayLevelNonUniformity
intra_original_glrlm_GrayLevelNonUniformity
intra_original_glrlm_RunEntropy
intra_original_glrlm_RunVariance
intra_original_glszm_GrayLevelNonUniformity
intra_original_glszm_LargeAreaEmphasis
intra_original_glszm_LargeAreaHighGrayLevelEmphasis
intra_original_glszm_SizeZoneNonUniformity
intra_original_glszm_SmallAreaHighGrayLevelEmphasis
intra_original_glszm_ZoneEntropy
intra_original_glszm_ZoneVariance
intra_original_shape_MajorAxisLength
intra_original_shape_Maximum2DDiameterColumn
intra_original_shape_Maximum2DDiameterRow
intra_original_shape_Maximum2DDiameterSlice
intra_original_shape_Maximum3DDiameter
intra_original_shape_MeshVolume
intra_original_shape_MinorAxisLength
intra_original_shape_Sphericity
intra_original_shape_SurfaceArea
intra_original_shape_VoxelVolume
