# Supplementary material for: Endoscopic ultrasonography-based intratumoral and peritumoral machine learning radiomics analyses for distinguishing insulinomas from non-functional pancreatic neuroendocrine tumors
Source: Front Endocrinol (Lausanne). 2024 Jun 17;15:1383814. doi: 10.3389/fendo.2024.1383814 (PMC11215175; doi:10.3389/fendo.2024.1383814)
Supplement: Supplementary file 2 [file DataSheet_2.pdf]

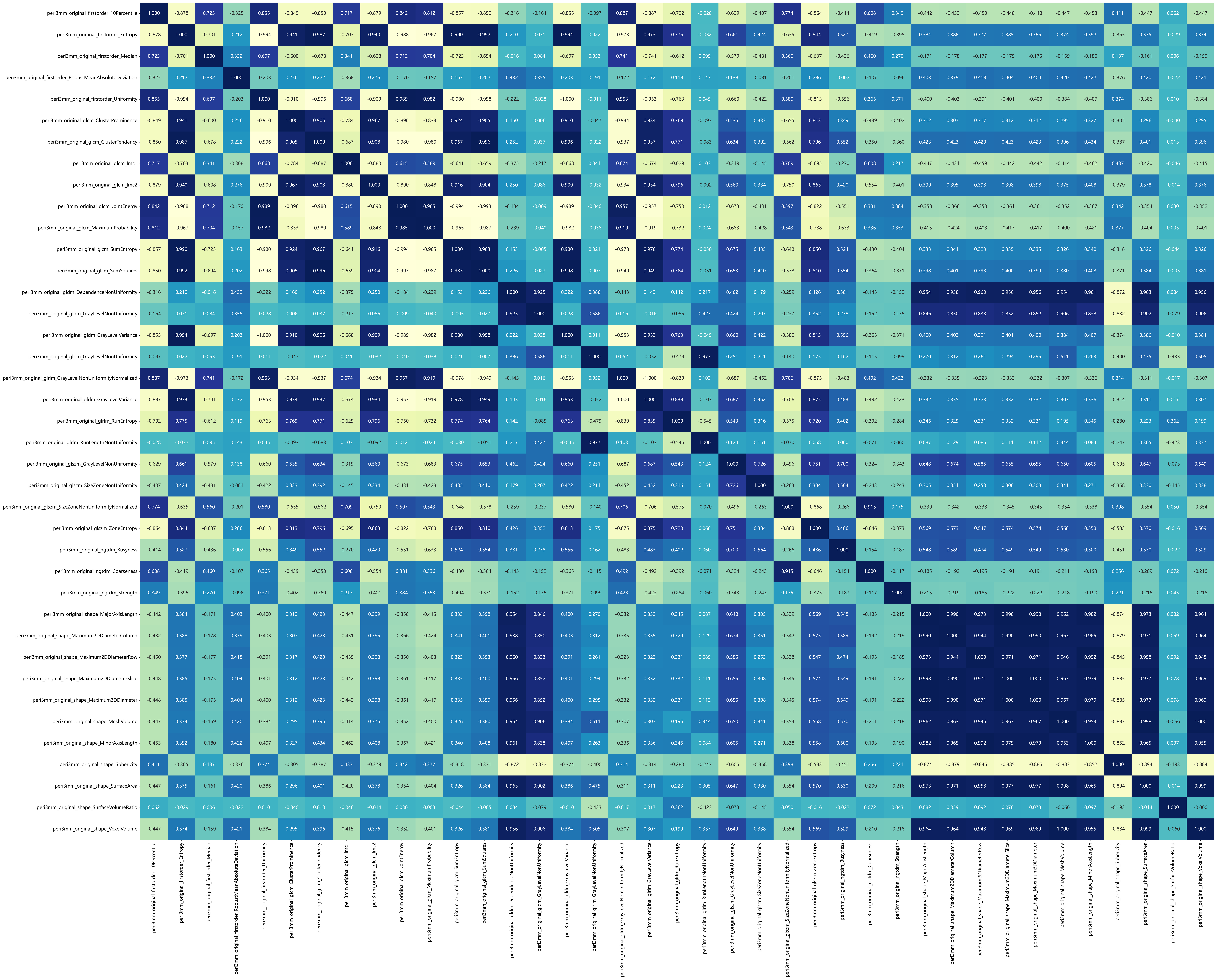
peri3mm_original_firstorder_10Percentile
peri3mm_original_firstorder_Entropy
peri3mm_original_firstorder_Median
peri3mm_original_firstorder_RobustMeanAbsoluteDeviation
peri3mm_original_firstorder_Uniformity
peri3mm_original_glcm_ClusterProminence
peri3mm_original_glcm_ClusterTendency
peri3mm_original_glcm_Imc1
peri3mm_original_glcm_Imc2
peri3mm_original_glcm_JointEnergy
peri3mm_original_glcm_MaximumProbability
peri3mm_original_glcm_SumEntropy
peri3mm_original_glcm_SumSquares
peri3mm_original_gldm_DependenceNonUniformity
peri3mm_original_gldm_GrayLevelNonUniformity
peri3mm_original_gldm_GrayLevelVariance
peri3mm_original_glrlm_GrayLevelNonUniformity
peri3mm_original_glrlm_GrayLevelNonUniformityNormalized
peri3mm_original_glrlm_GrayLevelVariance
peri3mm_original_glrlm_RunEntropy
peri3mm_original_glrlm_RunLengthNonUniformity
peri3mm_original_glszm_GrayLevelNonUniformity
peri3mm_original_glszm_SizeZoneNonUniformity
peri3mm_original_glszm_SizeZoneNonUniformityNormalized
peri3mm_original_glszm_ZoneEntropy
peri3mm_original_ngtdm_Busyness
peri3mm_original_ngtdm_Coarseness
peri3mm_original_ngtdm_Strength
peri3mm_original_shape_MajorAxisLength
peri3mm_original_shape_Maximum2DDiameterColumn
peri3mm_original_shape_Maximum2DDiameterRow
peri3mm_original_shape_Maximum2DDiameterSlice
peri3mm_original_shape_Maximum3DDiameter
peri3mm_original_shape_MeshVolume
peri3mm_original_shape_MinorAxisLength
peri3mm_original_shape_Sphericity
peri3mm_original_shape_SurfaceArea
peri3mm_original_shape_SurfaceVolumeRatio
peri3mm_original_shape_VoxelVolume
