# Supplementary material for: Endoscopic ultrasonography-based intratumoral and peritumoral machine learning radiomics analyses for distinguishing insulinomas from non-functional pancreatic neuroendocrine tumors
Source: Front Endocrinol (Lausanne). 2024 Jun 17;15:1383814. doi: 10.3389/fendo.2024.1383814 (PMC11215175; doi:10.3389/fendo.2024.1383814)
Supplement: Supplementary file 3 [file DataSheet_3.pdf]

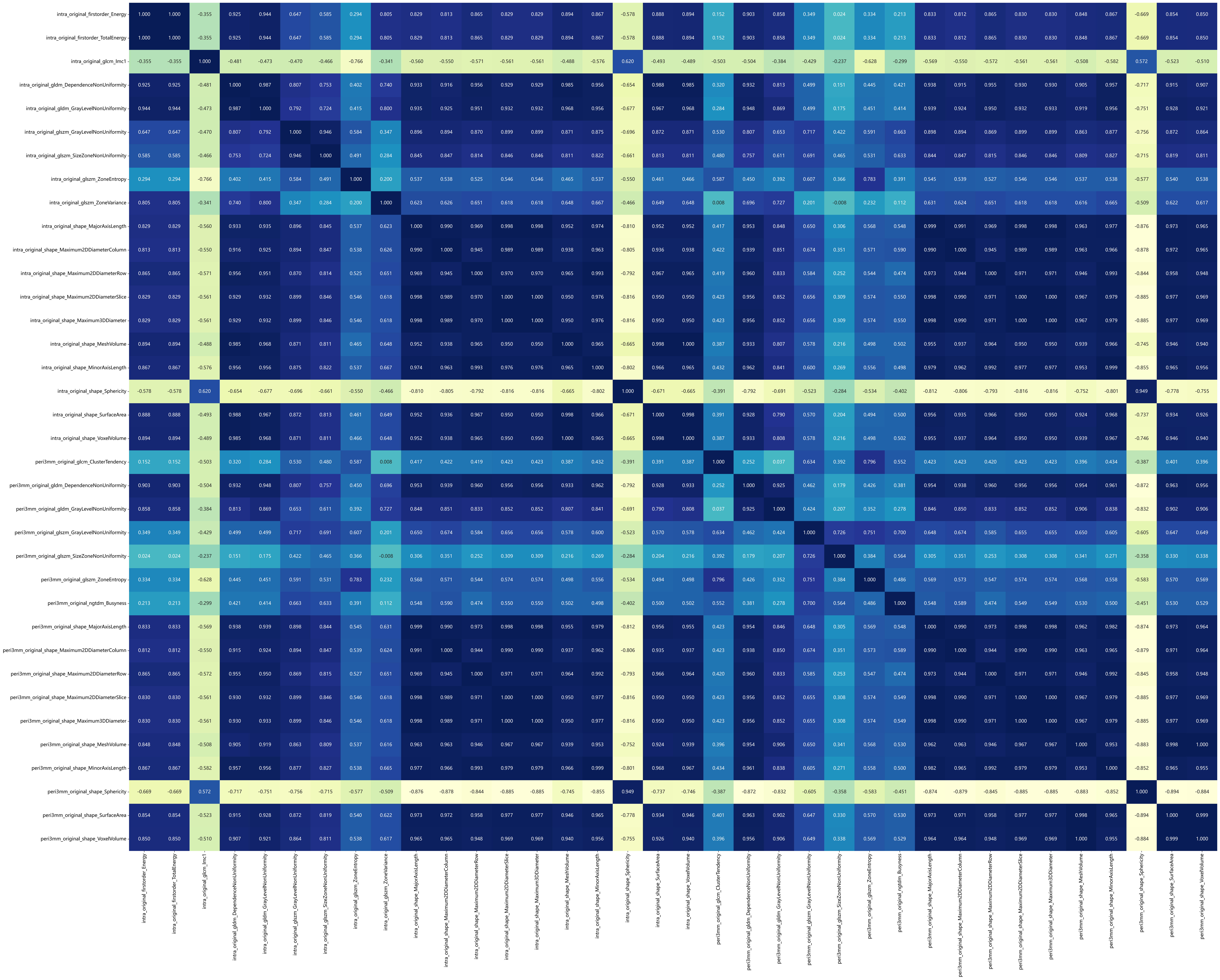
intra_original_firstorder_Energy
intra_original_firstorder_TotalEnergy
intra_original_glcm_Imc1
intra_original_gldm_DependenceNonUniformity
intra_original_gldm_GrayLevelNonUniformity
intra_original_glszm_GrayLevelNonUniformity
intra_original_glszm_SizeZoneNonUniformity
intra_original_glszm_ZoneEntropy
intra_original_glszm_ZoneVariance
intra_original_shape_MajorAxisLength
intra_original_shape_Maximum2DDiameterColumn
intra_original_shape_Maximum2DDiameterRow
intra_original_shape_Maximum2DDiameterSlice
intra_original_shape_Maximum3DDiameter
intra_original_shape_MeshVolume
intra_original_shape_MinorAxisLength
intra_original_shape_Sphericity
intra_original_shape_SurfaceArea
intra_original_shape_VoxelVolume
peri3mm_original_glcm_ClusterTendency
peri3mm_original_gldm_DependenceNonUniformity
peri3mm_original_gldm_GrayLevelNonUniformity
peri3mm_original_glszm_GrayLevelNonUniformity
peri3mm_original_glszm_SizeZoneNonUniformity
peri3mm_original_glszm_ZoneEntropy
peri3mm_original_ngtdm_Busyness
peri3mm_original_shape_MajorAxisLength
peri3mm_original_shape_Maximum2DDiameterColumn
peri3mm_original_shape_Maximum2DDiameterRow
peri3mm_original_shape_Maximum2DDiameterSlice
peri3mm_original_shape_Maximum3DDiameter
peri3mm_original_shape_MeshVolume
peri3mm_original_shape_MinorAxisLength
peri3mm_original_shape_Sphericity
peri3mm_original_shape_SurfaceArea
peri3mm_original_shape_VoxelVolume
