# Supplementary material for: Endoscopic ultrasonography-based intratumoral and peritumoral machine learning radiomics analyses for distinguishing insulinomas from non-functional pancreatic neuroendocrine tumors
Source: Front Endocrinol (Lausanne). 2024 Jun 17;15:1383814. doi: 10.3389/fendo.2024.1383814 (PMC11215175; doi:10.3389/fendo.2024.1383814)
Supplement: Supplementary file 4 [file DataSheet_4.pdf]

Variable	Overall, N = 106[1]	Insulinoma or NF-PNETs 0 N = 45 (42%)[1]	Insulinoma or NF-PNETs 1 N = 61 (58%)[1]	p-value[2]
**diameter**	16.00 [12.00, 28.90]	31.10 [22.00, 39.20]	12.00 [10.00, 15.80]	<0.001
**age**	49.00 [37.00, 57.00]	51.00 [37.00, 60.00]	48.00 [37.00, 56.00]	0.482
**location**				0.177
0	45 (42.45%)	23 (51.11%)	22 (36.07%)	
1	61 (57.55%)	22 (48.89%)	39 (63.93%)	
**cystic**				0.081
0	100 (94.34%)	40 (88.89%)	60 (98.36%)	
1	6 (5.66%)	5 (11.11%)	1 (1.64%)	
**calcification**				0.425
0	105 (99.06%)	44 (97.78%)	61 (100.00%)	
1	1 (0.94%)	1 (2.22%)	0 (0.00%)	
**uniformity**				0.082
0	52 (49.06%)	27 (60.00%)	25 (40.98%)	
1	54 (50.94%)	18 (40.00%)	36 (59.02%)	

[1] Median [IQR]; n (%)

[2] Wilcoxon rank sum test; Pearson’s Chi-squared test; Fisher’s exact test

**Variable**	**Overall**, N = 106[1]	**Insulinoma or NF-PNETs**<br>**0**<br>N = 45 (42%)[1]	**Insulinoma or NF-PNETs**<br>**1**<br>N = 61 (58%)[1]	**p-value**[2]
**echo**				0.261
0	15 (14.15%)	4 (8.89%)	11 (18.03%)	
1	91 (85.85%)	41 (91.11%)	50 (81.97%)	
**margin**				0.318
0	10 (9.43%)	6 (13.33%)	4 (6.56%)	
1	96 (90.57%)	39 (86.67%)	57 (93.44%)	
**shape**				0.197
0	34 (32.08%)	18 (40.00%)	16 (26.23%)	
1	72 (67.92%)	27 (60.00%)	45 (73.77%)	
**gender**				0.067
0	66 (62.26%)	23 (51.11%)	43 (70.49%)	
1	40 (37.74%)	22 (48.89%)	18 (29.51%)	

[1] Median [IQR]; n (%)

[2] Wilcoxon rank sum test; Pearson’s Chi-squared test; Fisher’s exact test
